# Life beneath the ice: jellyfish and ctenophores from the Ross Sea, Antarctica, with an image-based training set for machine learning

**DOI:** 10.3897/BDJ.9.e69374

**Published:** 2021-08-16

**Authors:** Gerlien Verhaegen, Emiliano Cimoli, Dhugal Lindsay

**Affiliations:** 1 Advanced Science-Technology Research (ASTER) Program, Institute for Extra-cutting-edge Science and Technology Avant-garde Research (X-star), Japan Agency for Marine-Earth Science and Technology (JAMSTEC), Yokosuka, Japan Advanced Science-Technology Research (ASTER) Program, Institute for Extra-cutting-edge Science and Technology Avant-garde Research (X-star), Japan Agency for Marine-Earth Science and Technology (JAMSTEC) Yokosuka Japan; 2 Institute for Marine and Antarctic Studies, College of Sciences and Engineering, University of Tasmania, Hobart, Australia Institute for Marine and Antarctic Studies, College of Sciences and Engineering, University of Tasmania Hobart Australia; 3 Discipline of Geography and Spatial Sciences, School of Technology, Environments and Design, College of Sciences and Engineering, University of Tasmania, Hobart, Australia Discipline of Geography and Spatial Sciences, School of Technology, Environments and Design, College of Sciences and Engineering, University of Tasmania Hobart Australia

**Keywords:** Southern Ocean, gelatinous zooplankton, siphonophore, video annotation, remotely-operated vehicle (ROV), Common Objects in Context (COCO), machine learning

## Abstract

**Background:**

Southern Ocean ecosystems are currently experiencing increased environmental changes and anthropogenic pressures, urging scientists to report on their biodiversity and biogeography. Two major taxonomically diverse and trophically important gelatinous zooplankton groups that have, however, stayed largely understudied until now are the cnidarian jellyfish and ctenophores. This data scarcity is predominantly due to many of these fragile, soft-bodied organisms being easily fragmented and/or destroyed with traditional net sampling methods. Progress in alternative survey methods including, for instance, optics-based methods is slowly starting to overcome these obstacles. As video annotation by human observers is both time-consuming and financially costly, machine-learning techniques should be developed for the analysis of *in situ* /*in aqua* image-based datasets. This requires taxonomically accurate training sets for correct species identification and the present paper is the first to provide such data.

**New information:**

In this study, we twice conducted three week-long *in situ* optics-based surveys of jellyfish and ctenophores found under the ice in the McMurdo Sound, Antarctica. Our study constitutes the first optics-based survey of gelatinous zooplankton in the Ross Sea and the first study to use *in situ / in aqua* observations to describe taxonomic and some trophic and behavioural characteristics of gelatinous zooplankton from the Southern Ocean. Despite the small geographic and temporal scales of our study, we provided new undescribed morphological traits for all observed gelatinous zooplankton species (eight cnidarian and four ctenophore species). Three ctenophores and one leptomedusa likely represent undescribed species. Furthermore, along with the photography and videography, we prepared a Common Objects in Context (COCO) dataset, so that this study is the first to provide a taxonomist-ratified image training set for future machine-learning algorithm development concerning Southern Ocean gelatinous zooplankton species.

## Introduction

Southern Ocean ecosystems have experienced increasing environmental changes over the last decades (Turner et al. 2014). These changes include ocean warming (e.g. Auger et al. 2021, Gille 2002, Sallée 2018), freshening (e.g. Rye et al. 2014, Swart et al. 2018), a poleward shift of ocean fronts (reviewed by Chapman et al. 2020), major ice shelf collapses (Ingels et al. 2021) and regional fluctuations in the extent and the seasonality of sea ice (e.g. Parkinson and Cavalieri 2012, Stammerjohn et al. 2008). Besides these environmental changes, the Southern Ocean has also been facing an increasing impact from economic activities, such as commercial fishing, tourism and scientific research (Ainley and Pauly 2014, Tin et al. 2009). These growing impacts, both environmental and anthropogenic, have hastened studies on Southern Ocean marine biodiversity and biogeography (e.g. De Broyer et al. 2014a), as well as investigations on how these changes may affect its marine biota, food webs and ecosystem services (e.g. Constable et al. 2014, Smetacek and Nicol 2005). For instance, sea ice plays a key role in controlling primary production; hence, fluctuations in its coverage can trigger cascading effects at multiple marine food web levels (Massom and Stammerjohn 2010). Although one major gelatinous zooplankton taxon, the salps, have been extensively studied (e.g. Atkinson et al. 2004, Kawaguchi et al. 2004, Plum et al. 2020), two major gelatinous zooplankton groups have, however, stayed largely understudied until now: cnidarian jellyfish (i.e. siphonophores, hydromedusae and scyphomedusae, referred to hereafter as "jellyfish") and ctenophores.

Gelatinous zooplankton, comprising jellyfish, ctenophores and chordate tunicates (Pagès 1997), are ubiquitous in the oceans and can occur in large blooms (Richardson et al. 2009, Schaub et al. 2018). Compared to commercially relevant marine species (cf. FishBase and SeaLifeBase) or hard-bodied zooplankton, such as copepods and krill (e.g. Schnack-Schiel and Mujica 1994, Smith and Schnack-Schiel 1990), gelatinous zooplankton data are critically scarce and lack a reliable baseline (Pauly et al. 2009). This data scarcity is due to three major reasons, the first being that these fragile, soft-bodied organisms are easily fragmented and/or destroyed with traditional net sampling (Licandro et al. 2015). Secondly, gelatinous zooplankton, especially ctenophores, are extremely difficult to chemically fix and preserve (e.g. Engell-Sørensen et al. 2009, Mutlu 1996, Thibault-Botha and Bowen 2004). As a result, accurate taxonomic descriptions and morphological measurements require processing the specimens upon collection or using live material (e.g. Deason 1982, Lindsay et al. 2017). Furthermore, because of deformed, shrunken or completely disintegrated holotypes and paratypes, taxonomic comparisons can often only rely on the original description and drawings of species (e.g. Haeckel 1879, Moser 1909). A final reason as to why gelatinous zooplankton have been largely overlooked is because, historically, their ecological roles have often been ignored. Indeed, despite being well-known predators, gelatinous zooplankton have long been considered trophic dead ends (Verity and Smetacek 1996). A paradigm shift in the recognition of their trophic importance has, however, occurred after new approaches for studying diet revealed that a wide and diverse range of marine predators, including fish, birds, turtles and invertebrates (e.g. octopus, sea cucumbers, crabs and amphipods) consume gelatinous zooplankton (reviewed by Hays et al. 2018).

Recently, the drawbacks in the collection and identification of gelatinous zooplankton have slowly started to be overcome through progress in methodologies using, for instance, molecular tools (e.g. metabarcoding, environmental DNA: Lindsay et al. 2017, Minamoto et al. 2017, Questel et al. 2021, Bucklin et al. 2021), acoustics (e.g. Båmstedt et al. 2003, Brierley et al. 1991, Zhang et al. 2019) and optics [e.g. Remotely Operated Vehicle (ROV) and Underwater Video Profiler surveys: Ford et al. 2020, Hidaka et al. 2021, Hosia et al. 2017]. In the case of the latter, the time-consuming and financially costly process to annotate videos by human observers has created the need to develop automated techniques (Caughlan and Oakley 2001, Del Vecchio et al. 2019). Machine learning is one of these techniques, in which a computer system learns patterns from a training dataset and then subsequently can find these same patterns in another independent test dataset. The first study using machine learning to classify plankton images dates back to 1980, for which pattern extraction on digital microscopy images to classify five genera of phytoplankton was performed (Schlimpert et al. 1980). Since then, machine-learning techniques for image and video annotation of plankton have been drastically improved and a significant increase in published papers was observed after 2012 (reviewed by Irisson et al. In press). The use of machine learning for image and video annotation of gelatinous zooplankton remains, however, scarce and most of these first studies could not differentiate between jellyfish species (Kim et al. 2016, Rife and Rock 2003). The few image-based machine-learning studies that could differentiate between some jellyfish species included the detection of moon jellyfish through underwater sonar imagery (French et al. 2019) and a real-time jellyfish monitoring tool for three Mediterranean jellyfish species using a deep learning object detection-based neutral network (Martin-Abadal et al. 2020). As the future of studying gelatinous zooplankton through *in situ* optical methods certainly lies in the development of more efficient and accurate video/image analysis tools, with machine-learning-based algorithms able to distinguish between the numerous species, an additional difficulty is providing an accurate training dataset. As biogeographic datasets and imaging libraries for gelatinous zooplankton are growing, species misidentification is not uncommon, highlighting the need for taxonomically-accurate datasets (Lindsay et al. 2017).

Surveys of gelatinous zooplankton in the Southern Ocean flourished in the late 19^th^ and early 20^th^ centuries. These surveys were conducted during famous expeditions, such as the *Gauss* expedition 1901–1903 (that is the first German expedition to Antarctica, also known as the “Deutsche Südpolar-Expedition 1901–1903”) (e.g. Moser 1909, Moser 1925, Vanhöffen 1912), the British Southern Cross 1898-1900 and Discovery 1901–1904 expeditions (e.g. Browne 1910), the Scottish *Scotia* expedition 1902–1904, the Belgian *Belgica* 1897–1899 expedition (i.e. first expedition to overwinter in the Antarctic Region), the French *Français* expedition 1904–1907 and the Swedish *Antarctic* 1901–1903 expedition (i.e. considerable parts of the collected material were lost after the ship was crushed by the ice and sank) (Kramp 1948). After this so-called Heroic Age of Antarctic Exploration, surveys remained sporadic (e.g. Kramp 1949, Kramp 1957b), up until the cruises of the USNS *Eltanin* between 1962 and 1972 (Larson 1986, Navas-Pereira and Vannucci 1990, Alvarino et al. 1990). Totalling up to 52 Antarctic research cruises, the USNS *Eltanin* records still account today for the majority of the occurrence data for gelatinous zooplankton in the Southern Ocean, concentrated south of 35°S, between the longitudes of 20-130°W (Lindsay et al. 2014). In more recent years, noteworthy Southern Ocean campaigns targeting these elusive animals included, but were not limited to the *Antarktis* cruises of the R/V *Polarstern* (e.g. Pagès and Kurbjeweit 1994, Pagès and Schnack-Schiel 1996, Pagès and Pugh 2002) and cruises of the R/V *Umitaka-Maru* (e.g. Grossmann et al. 2013, Grossmann et al. 2012, Toda et al. 2014). All these surveys were conducted through net-sampling and, although surveys continue to be conducted up to this day, this has remained the major, nearly exclusive, sampling method [e.g. through MOCNESS, Multinet, NORPAC net, Rectangular Midwater Trawl (RMT) etc.Grossmann 2010, Kaufmann et al. 2011, Lindsay et al. 2014].

Reports employing alternative survey methods for gelatinous zooplankton in the Southern Ocean, such as genetics or optics-based surveys, are few. For instance, sequences suitable for DNA barcoding remain rare, especially at species-level taxonomic resolution, with the notable exceptions of some siphonophore species (e.g. Lindsay et al. 2015, Panasiuk et al. 2018) and a few scattered medusa sequences (e.g. Collins et al. 2008, Schuchert 2017). Although not directly from pelagic specimens, more DNA barcoding efforts have been conducted on benthic hydroids, at least some of which also possess a medusa stage (e.g. Cantero et al. 2010, Maronna et al. 2016). The scarce optical surveys include the use of: hand-collection in jars (Larson and Harbison 1990) and underwater photographs (Brueggeman 1998) by SCUBA, video plankton recorder (VPR) for larvaceans (Lindsay 2012), autonomous visual plankton recorder (AVPR) (Lindsay 2010), a ROV in close proximity to icebergs (Sherlock et al. 2011) and even video loggers placed on penguins (Thiebot et al. 2016, Thiebot et al. 2017). Adding to this short list, we have performed a much-needed optics-based survey by filming gelatinous zooplankton from under the ice in the Ross Sea, Antarctica. To our knowledge, this is the first study to use *in situ* and live animal-based photography and videography to describe taxonomic and some trophic and behavioural characteristics of living hydromedusae, scyphomedusae, siphonophores and ctenophores from the Southern Ocean. Furthermore, we prepared a Common Objects in Context (COCO) dataset, so that this study is the first to provide a taxonomist-ratified image training set for future machine-learning algorithm development concerning Southern Ocean gelatinous zooplankton species.

## Materials and methods

### Study location

**Figure 1.**
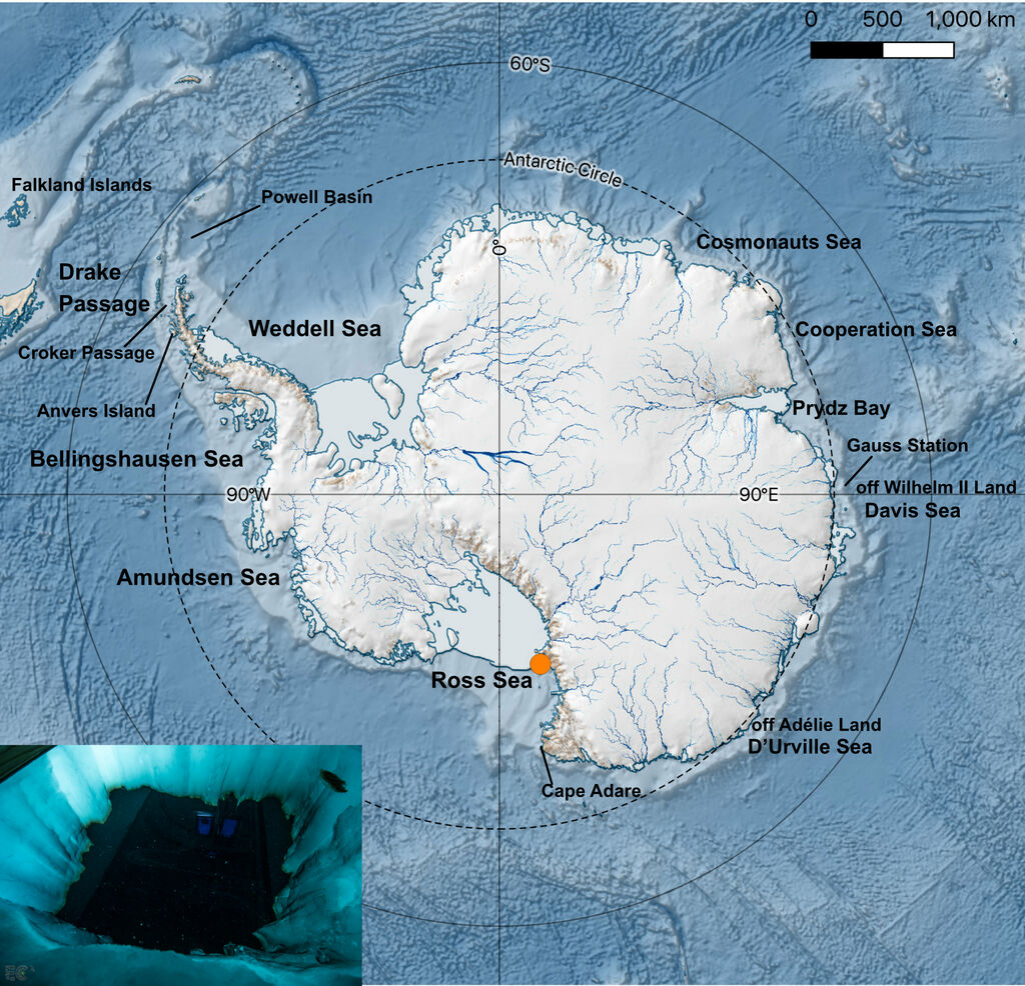
Location (orange dot) and picture (insert) of the 2 × 1.8 m ice-hole from which the *in situ* /*in aqua* imagery and video data were taken during this study.

Imagery and video data of under-ice gelatinous zooplankton were acquired at Cape Evans (McMurdo Sound, Ross Sea) over two different field campaigns conducted during the period of November-December 2018 and 2019 (Antarctic summer). A field camp was established for a duration of 3 weeks for each campaign, and was located approximately 200 m from the coast on Antarctic fast-ice (77.637° S, 166.401°E) (Fig. 1). The seabed topography beneath the field camp consisted of a slope with a water depth ranging from 10 to 17 m. During spring, Cape Evans is characterised by a relatively homogenous fast-ice cover, the thickness of which was 1.8 ± 0.02 m in 2018 and 1.3 ± 0.05 m for 2019. The sea-ice surface in the area typically features a snow-free landscape, induced by strong winds and a featureless topography. The fast-ice of Cape Evans and, in general, of the entire coastal Antarctic fast-ice ecosystem, is known to be highly productive and rich in ice algal biomass (Cimoli et al. 2019, Arrigo 2017). Due to the proximity of the American McMurdo Research Station, various net surveys of gelatinous zooplankton have been conducted in this area in the past (e.g. Foster 1989, Larson and Harbison 1990, Browne 1910). However, this study represents the first optics-based survey of gelatinous zooplankton in the Ross Sea.

### Photography and videography

For both campaigns, a large 2 × 1.8 m ice-hole was made through a combination of 6” Jiffy auger holes and hot-water drilling (Fig. 1). A polar haven tent was erected on top of the hole providing a relatively dark imaging studio, where subjects could be imaged under controlled illumination conditions. Diverse under-ice gelatinous zooplankton naturally approached the large ice hole surface during different times of the day and the diversity of subjects was constantly being documented through high-resolution macrophotography and videography. A Sony Alpha 7 III camera, equipped with a FE 90mm F2.8 Macro G OSS lens, was used for this purpose. The camera was mounted on a standard underwater camera tray fitted with an underwater strobe arm and a Light & Motion Sola 2500F LED video light that provided the power of 2500 lumen directed in a floodlight (60° beam pattern). Most of the imagery and videography were acquired with the subjects *in situ* as they neared the water-air interface and the LED light immersed in the water. Subjects smaller in size or dynamic in movement, were scooped out of the water using a 15 × 15 cm acrylic glass container filled with water at the *in situ* temperature, imaged with the same set-up under controlled illumination conditions and then returned to the water. It is worth mentioning that this observation methodology was non-exhaustive and limited to organisms that were easily seen from the ice-hole opening.

Underwater footage of the entire study area was conducted using two different Remotely Operated Vehicles (ROVs), equipped with a GoPro Hero 5; a Seabotix LBV-300 ROV (Teledyne Marine, California, USA) for the 2018 campaign and a BlueROV2 (Blue Robotics, California, USA) for the 2019 campaign. Additional underwater footage straight beneath the ice hole was acquired using Boxfish 360’s three large Micro Four Thirds cameras (Boxfish Research Limited, Auckland, New Zealand) deployed at different depths of the water columns using a weighted rope.

### Treatment of images and videos

The raw, untreated images and videos were used to build online datasets (see "Data resources" section). The brightness and contrast of the images to build the plates (Figs. 2-19) were sometimes altered to reveal underlying morphological structures. The Common Objects in Context (COCO) dataset was generated by annotating the specimens in the images and videos using the free, open source, Computer Vision Annotation Tool (CVAT) (https://github.com/openvinotoolkit/cvat). COCO is a large-scale object detection, segmentation and captioning dataset. It is the most popular type of dataset used for training deep learning programmes.

## Data resources

The occurence data reported in this paper are deposited at GBIF, the Global Biodiversity Information Facility, http://ipt.pensoft.net/resource?r=life_beneath_the_ice-jellyfish_and_ctenophores_from_the_ross_sea_antarctica&amp. The raw, untreated images and videos are available at http://morphobank.org/permalink/?P3993 and https://www.youtube.com/playlist?list=PL5Njywnb4yMJQa7koLOM3BhjKU7Ii2HiZ, respectively. The COCO datasets can be found on https://zenodo.org/ with the following DOI:10.5281/zenodo.5118013.

## Checklists

### Anthoathecata

#### Koellikerina maasi

(Browne, 1910)

FDF620AB-2967-50F2-A3A3-916F38CF394E

##### Materials

- **Type status:**Other material. **Occurrence:** individualID: MCMEC2019_Koellikerina_maasi_a; lifeStage: adult; associatedMedia: "http://morphobank.org/permalink/?P3993", "https://youtu.be/QiBPf_HYrQ8", "https://youtu.be/-BonvTRljY8"; **Taxon:** scientificName: Koellikerinamaasi; kingdom: Animalia; phylum: Cnidaria; class: Hydrozoa; order: Anthoathecata; family: Bougainvilliidae; genus: Koellikerina; **Location:** continent: Antarctica; waterBody: McMurdo Sound; maximumDepthInMeters: 1; decimalLatitude: -77.637; decimalLongitude: 166.401; **Identification:** identifiedBy: Dhugal Lindsay; **Event:** samplingProtocol: Sony Alpha 7 III camera equipped with a FE 90mm F2.8 Macro G OSS lens; eventDate: 2019-11-26; **Record Level:** type: StillImage, Video; language: en; rightsHolder: Emiliano Cimoli
- **Type status:**Other material. **Occurrence:** individualID: MCMEC2018_Koellikerina_maasi_b; lifeStage: adult; associatedMedia: http://morphobank.org/permalink/?P3993; **Taxon:** scientificName: Koellikerinamaasi; kingdom: Animalia; phylum: Cnidaria; class: Hydrozoa; order: Anthoathecata; family: Bougainvilliidae; genus: Koellikerina; **Location:** continent: Antarctica; waterBody: McMurdo Sound; maximumDepthInMeters: 1; decimalLatitude: -77.637; decimalLongitude: 166.401; **Identification:** identifiedBy: Dhugal Lindsay; **Event:** samplingProtocol: NIKON D500 camera equipped with a TAMRON SP 90mm F2.8 Di Macro VC USD F017N lens; eventDate: 2018-11-27; **Record Level:** type: StillImage; language: en; rightsHolder: Emiliano Cimoli
- **Type status:**Other material. **Occurrence:** individualID: MCMEC2018_Koellikerina_maasi_c; lifeStage: adult; associatedMedia: http://morphobank.org/permalink/?P3993; **Taxon:** scientificName: Koellikerinamaasi; kingdom: Animalia; phylum: Cnidaria; class: Hydrozoa; order: Anthoathecata; family: Bougainvilliidae; genus: Koellikerina; **Location:** continent: Antarctica; waterBody: McMurdo Sound; maximumDepthInMeters: 1; decimalLatitude: -77.637; decimalLongitude: 166.401; **Identification:** identifiedBy: Dhugal Lindsay; **Event:** samplingProtocol: NIKON D500 camera equipped with a TAMRON SP 90mm F2.8 Di Macro VC USD F017N lens; eventDate: 2018-11-29; **Record Level:** type: StillImage; language: en; rightsHolder: Emiliano Cimoli

##### Distribution

Southern Ocean, in the McMurdo Sound (Browne 1910, Foster 1989, Larson and Harbison 1990), off Adélie Land (Toda et al. 2014), off Wilhelm II Land at Gauss Station (66.03°S, 89.63°E) (Vanhöffen 1912), in Prydz Bay (Hosie 1999a, Hosie 1999b) and in the Weddell Sea (Kramp 1957a); New Zealand (Bouillon 1995, Schuchert 1996); Madagascar (Kramp 1965); Papua New Guinea (Bouillon et al. 1988).

##### Notes

**Figure 2.**
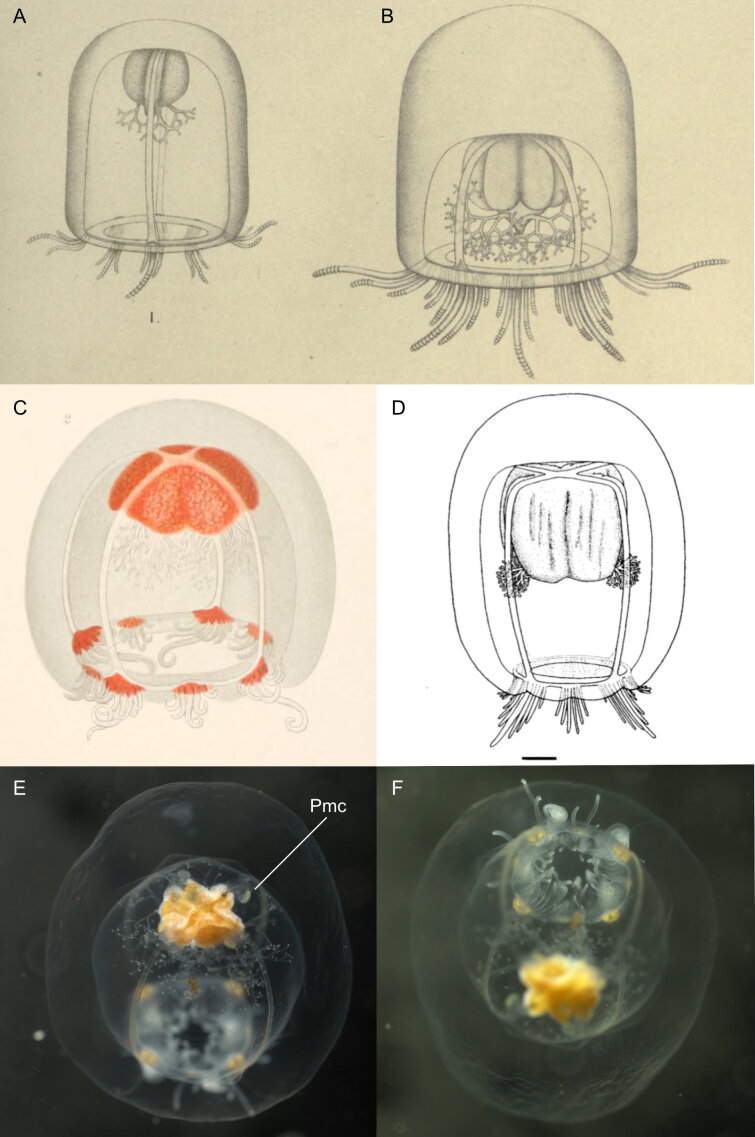
*Koellikerinamaasi*. **A, B.** Drawing from the original species description by Browne (1910) of a specimen in an early developmental stage (A) and an adult specimen (B) from McMurdo Sound, Antarctica; **C.** Drawing of a specimen (bell height 11 mm) from Gauss Station, Antarctica (Vanhöffen 1912); **D.** Drawing of a specimen from New Zealand (Schuchert 1996, Suppl. material 1), scale bar 1 mm; **E, F.** Specimen MCMEC2019_Koellikerina_maasi_a observed on 26/11/2019; apico-lateral (E) and oral-lateral (F) views. Pmc: perradial mesogleal convexity with ovoid yellowish nodule. E-F photos courtesy: E. Cimoli.

Original description afterBrowne (1910)(basionym *Koellikeriamaasi* Browne, 1910) (Fig. 2A-B): Bell-shaped medusa, with very thick, higher than broad (up to 9 mm wide and 10 mm high), umbrella with a rounded summit; four broad radial canals, adjacent to the ectodermal lining of the sub-umbrella, attached at the base of the stomach; radial grooves in the wall of the sub-umbrella, adjacent to the radial canals; large and cross-shaped stomach, interior covered with minute endodermal papillae, with a slender mesogleal strand running along the centre of the papilla; four dichotomously branched perradial oral tentacles inserted above the mouth rim, the number of branches increasing with age (two-three times dichotomously branched for young stage, at least seven times branched in adult stage), distal branches terminating with small nematocyst-covered cap; mouth circular and simple; four perradial gonadal masses, covering nearly entirely the outer wall of the manubrium; eight groups of solid marginal tentacles (four perradial and four interradial), the number of tentacles in each group increasing with age, with three to seven tentacles in the perradial groups and three to five tentacles in the interradial groups, the middle tentacle per group being the longest, with the middle tentacle of the perradial groups being longer than the middle one of the interradial groups; no ocelli present. Characters gleaned from species’ illustrations (for which the adult drawing was based on several specimens): mesogleal thickness between the ex- and sub-umbrella on the top of the bell ca. one fourth of the height of the ex-umbrella in young specimens and ca. half the height in adults; manubrium size ca. one third the height of the sub-umbrella in young specimens, ca. half the height in adults. Type locality: McMurdo Sound (78°49’S, 166°20’E), Antarctica.

Additional information from specimens from the Southern Ocean: from **Gauss Station** (0-385 m depth) (Vanhöffen 1912) (Fig. 2C), adult specimen preserved in formalin, 11 mm high × 10 mm wide, sub-umbrella 7 mm high × 8 mm wide, mesogleal thickness between the ex- and sub-umbrella on top of the bell of 4 mm (ca. one third of the bell height), stomach not on peduncle (i.e. “Magenstiel” in the German original version) 2.5 mm high × 4 mm wide, gonads separated perradially and folded interradially, stomach and tentacle bulbs red in living specimens, but turned yellow once preserved in formalin, no ocelli (i.e. “Ozellen”), oral tentacles five times dichotomously branched, perradial tentacle bulbs with seven marginal tentacles, with middle tentacle the longest (ca. three times longer and thicker than the surrounding second-largest tentacles), interradial bulbs with five dissimilar tentacles. Young specimen of 1 mm in length, beginning of mouth-tentacles present in little buds at the perradial mouth corners, colour of four perradial tentacle bulbs, stomachs and radial canals yellow, perradial tentacle bulbs with one middle-sized and two smaller tentacles and two weak indications of additional tentacles, four smaller interradial tentacle groups consisting of three tentacles and without indications of two additional tentacles; **Weddell Sea** (Kramp 1957a), description matching the original one of Browne (1910).

Additional information on specimens identified as same species from outside the Southern Ocean: from west coast of **Madagascar** (Kramp 1965), diameter 8 mm, height 9 mm, slight indication of a gastral peduncle, description otherwise matching with Browne (1910), Vanhöffen (1912) and Kramp (1957a); from **Papua New Guinea** (Bouillon et al. 1988), gastric endoderm showing villi (also found in *K.constricta*, *K.fasciculata*, *K.octonemalis* and *K.ornata*); from **New Zealand** (Schuchert 1996) (Fig. 2D), two specimens examined, 9 mm diameter, description similar to Browne (1910), except for the gonads, that formed irregular vertical folds, which may have been caused by the fixation.

Literature giving diagnostic characters without describing new specimens:Bouillon (1995), Kramp (1961), Kramp (1968), O'Sullivan (1982).

Description and comments on observed material (Fig. 2E-F): N = 2 in 2018, N = 1 in 2019.

New undescribed characteristics: Ex-umbrella not smooth, showing small concavities and warts; the radial canals departing from the manubrium bend downwards, extending over four small perradial mesogleal convexities with ovoid yellowish nodules, before bending back up again to run over the ectodermal cavity of the sub-umbrella to the bell rim. These perradial mesogleal convexities are similar to those seen in the Leptothecate medusa *Modeeriarotunda* Quoy & Gaimard, 1827 (Pagès et al. 2006).

Characteristics differing from previous descriptions: mesogleal thickness between the ex- and sub-umbrella on the top of the bell ca. one fourth of the height of the ex-umbrella, similar to the drawing of the New Zealand specimen of Schuchert (1996) (Fig. 2D), but narrower compared to the descriptions by Browne (1910) and Vanhöffen (1912); manubrium size ca. one third of the height of the sub-umbrella, whereas ca. half the height for Browne (1910); triangular tentacular bulbs with tentacles arranged linearly as described by Browne (1910), whereas the diagnosis of Kramp (1968) mentions triangular bulbs in the text, but the dichotomous key (p. 35) reports them to be linear. We ascertain, based on the present live material, that they are indeed triangular.

#### Leuckartiara brownei

Larson & Harbison, 1990

E4C9F4C2-45E0-5289-AF05-44D91798F0DA

##### Materials

- **Type status:**Other material. **Occurrence:** individualID: MCMEC2019_Leuckartiara_brownei_a; lifeStage: adult; associatedMedia: "http://morphobank.org/permalink/?P3993", "https://youtu.be/QkFIkgJPmto", "https://youtu.be/fRwpi5KAhWQ", "https://youtu.be/dEIbVYlF_TQ", "https://youtu.be/liqjNkGn3Sk"; **Taxon:** scientificName: Leuckartiarabrownei; kingdom: Animalia; phylum: Cnidaria; class: Hydrozoa; order: Anthoathecata; family: Pandeidae; genus: Leuckartiara; **Location:** continent: Antarctica; waterBody: McMurdo Sound; maximumDepthInMeters: 1; decimalLatitude: -77.637; decimalLongitude: 166.401; **Identification:** identifiedBy: Dhugal Lindsay; **Event:** samplingProtocol: Sony Alpha 7 III camera equipped with a FE 90mm F2.8 Macro G OSS lens; eventDate: 2019-11-16; **Record Level:** type: StillImage, Video; language: en; rightsHolder: Emiliano Cimoli
- **Type status:**Other material. **Occurrence:** individualID: MCMEC2018_Leuckartiara_brownei_b; lifeStage: adult; associatedMedia: http://morphobank.org/permalink/?P3993; **Taxon:** scientificName: Leuckartiarabrownei; kingdom: Animalia; phylum: Cnidaria; class: Hydrozoa; order: Anthoathecata; family: Pandeidae; genus: Leuckartiara; **Location:** continent: Antarctica; waterBody: McMurdo Sound; maximumDepthInMeters: 1; decimalLatitude: -77.637; decimalLongitude: 166.401; **Identification:** identifiedBy: Dhugal Lindsay; **Event:** samplingProtocol: NIKON D500 camera equipped with a TAMRON SP 90mm F2.8 Di Macro VC USD F017N lens; eventDate: 2018-11-29; **Record Level:** type: StillImage; language: en; rightsHolder: Emiliano Cimoli
- **Type status:**Other material. **Occurrence:** individualID: MCMEC2018_Leuckartiara_brownei_c; lifeStage: adult; associatedMedia: http://morphobank.org/permalink/?P3993; **Taxon:** scientificName: Leuckartiarabrownei; kingdom: Animalia; phylum: Cnidaria; class: Hydrozoa; order: Anthoathecata; family: Pandeidae; genus: Leuckartiara; **Location:** continent: Antarctica; waterBody: McMurdo Sound; maximumDepthInMeters: 1; decimalLatitude: -77.637; decimalLongitude: 166.401; **Identification:** identifiedBy: Dhugal Lindsay; **Event:** samplingProtocol: NIKON D500 camera equipped with a TAMRON SP 90mm F2.8 Di Macro VC USD F017N lens; eventDate: 2018-11-29; **Record Level:** type: StillImage; language: en; rightsHolder: Emiliano Cimoli

##### Distribution

Southern Ocean, in the McMurdo Sound [described by Browne (1910) as a juvenile *Perigonimus* sp. according to Larson and Harbison (1990), Browne (1910)], off Adélie Land (Toda et al. 2014), in the Weddell Sea (Grossmann 2010, Pagès and Schnack-Schiel 1996) and in the Powell Basin (Kaufmann et al. 2011). It was also reported from the Mediterranean by Bouillon et al. (2000).

##### Notes

**Figure 3.**
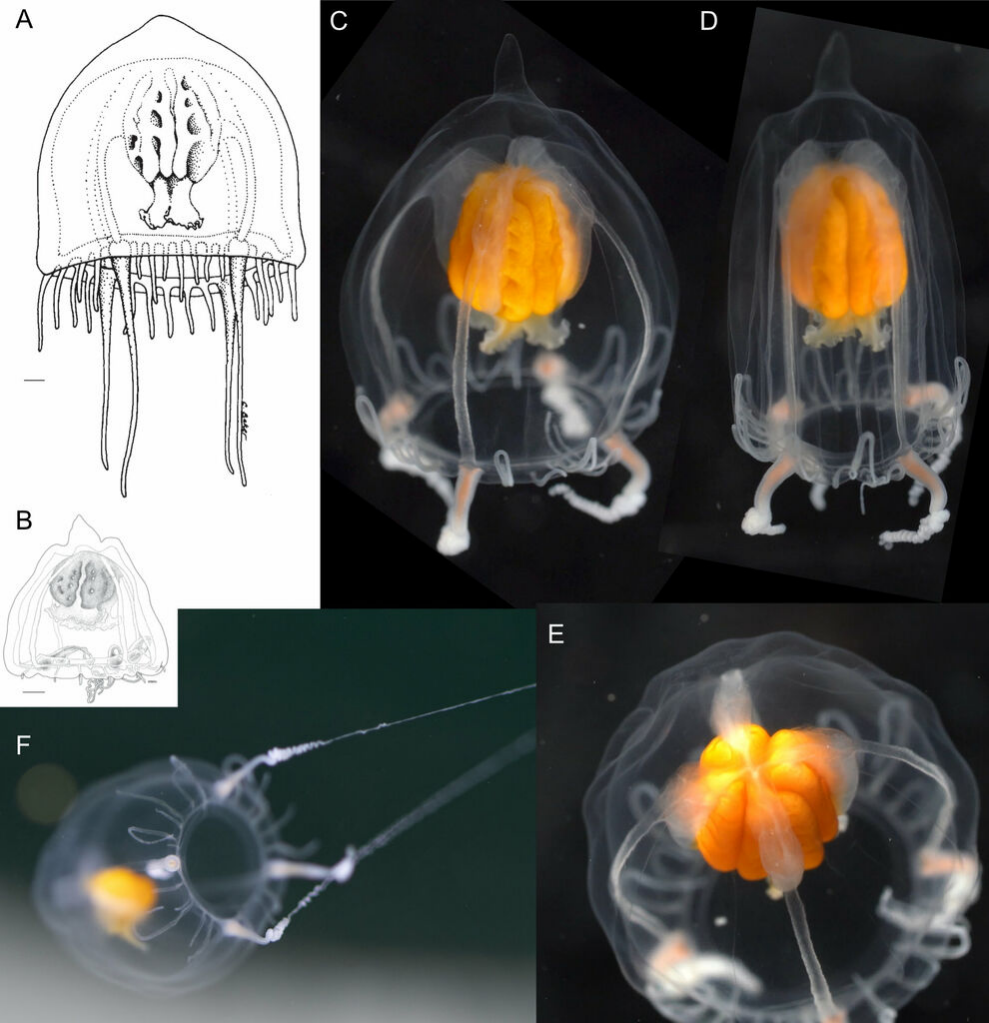
*Leuckartiarabrownei*. **A.** Line drawing of the holotype (bell height 10 mm) (Larson and Harbison 1990, Suppl. material 2) sampled in McMurdo Sound, Antarctica; **B.** Line drawing from a specimen (bell height 7 mm) from the Mediterranean Sea (modified from Bouillon et al. 2000); **C-E.** Specimen MCMEC2019_Leuckartiara_brownei_ a observed on 16/11/2019: lateral view (C), lateral view while contracting and apical view (E); **F.** Oral-lateral view of a specimen MCMEC2019_Leuckartiara_brownei_b observed on 29/11/2018. Scale bars A and B 1 mm. C-E photos courtesy: E. Cimoli.

Original description afterLarson and Harbison (1990)(Fig. 3A): Conical umbrella with a pointed projection of variable height (dimensions of holotype: 10 mm high × 9 mm wide); thick mesoglea; velum narrow, thin, and transparent; four large perradial tentacles, tapering and not laterally compressed, which are coiled when contracted; salmon-coloured perradial tentacle bulbs; up to 28 short “rudimentary” tentacles (i.e. because they have the same form as marginal tentacles in their early development stage; Russell 1953), growing in succession and clasping the ex-umbrella [*sic*: being clasped by the ex-umbrella], with the oldest interradial tentacles extending the furthest out on to the ex-umbrella; relatively large manubrium, with the height larger than half the height of the sub-umbrella; mesenteries well-developed; large crenulated lips; orange-brown gonads, covering the interradial surface of the manubrium, each gonad harbouring a pair of longitudinal folds adjacent to the interradii, forming a continuous interradial groove, with a few isolated folds in the adradii which are mostly orientated perradially; no ocelli or spurs. Type locality: near McMurdo Station, Antarctica.

Additional information on specimens identified as same species from outside the Southern Ocean: from the **Mediterranean Sea** (Bouillon et al. 2000) (Fig. 3B), height 7 mm, specimen not further described, but drawing available.

Literature giving diagnostic characters without describing new specimens:Pagès et al. (1992), Schuchert (2007), Bouillon et al. (2004).

Description of and comments on observed material (Fig. 3C-F): N = 2 in 2018, N = 1 in 2019. The morphology of our observed specimens matched closely those from previous descriptions. The height of the apical pointed projection ca. 15-20% of the bell height, corresponding to the “variable height” from the original description (Browne 1910). Number of gonadal folds 4-5 within the same specimen. Differences or additional information found compared to past descriptions were the following: the mesenteries extended to ca. 80% of the stomach height, whereas, in the original description (Browne 1910), they were described as “well developed” and shown to extend to ca. half the height of the stomach in the line drawing (Fig. 3A), while in Bouillon et al. (2000), the mesenteries were omitted from the drawing (Fig. 3B); coiling of the four main tentacles; the rudimentary tentacles, when of a certain length, can fold in half, with the distal half of the tentacle extended back downwards on to the ex-umbrella and the fold reaching up to 1/4 of the bell height, suggesting they are adnate to around half their length.

### Narcomedusae

#### Solmundella bitentaculata

(Quoy & Gaimard, 1833)

B8139797-14CE-5599-9F80-7BBBE4F716DB

##### Materials

- **Type status:**Other material. **Occurrence:** individualID: MCMEC2018_Solmundella_bitentaculata_a; lifeStage: adult; associatedMedia: http://morphobank.org/permalink/?P3993; **Taxon:** scientificName: Solmundellabitentaculata; kingdom: Animalia; phylum: Cnidaria; class: Hydrozoa; order: Narcomedusae; family: Solmundaeginidae; genus: Solmundella; **Location:** continent: Antarctica; waterBody: McMurdo Sound; maximumElevationInMeters: 1; decimalLatitude: -77.637; decimalLongitude: 166.401; **Identification:** identifiedBy: Dhugal Lindsay; **Event:** samplingProtocol: NIKON D500 camera equipped with a TAMRON SP 90mm F2.8 Di Macro VC USD F017N lens; eventDate: 2018-11-27; **Record Level:** type: StillImage; language: en; rightsHolder: Emiliano Cimoli

##### Distribution

Cosmopolitan (OBIS 2020). In the Southern Ocean: in the McMurdo Sound (Foster 1989, Larson and Harbison 1990), in the Bellingshausen Sea (Kramp 1957a), Croker Passage (Panasiuk-Chodnicka and Żmijewska 2010), in the Weddell Sea (Pagès and Schnack-Schiel 1996, Grossmann 2010), in Prydz Bay (Hosie 2012, Hosie 1999c, Hosie 1999a), off Adélie Land (Toda et al. 2014) and eastern Southern Ocean (south of 35°S, between 15°W and 160°E) (Navas-Pereira and Vannucci 1990).

##### Notes

**Figure 4.**
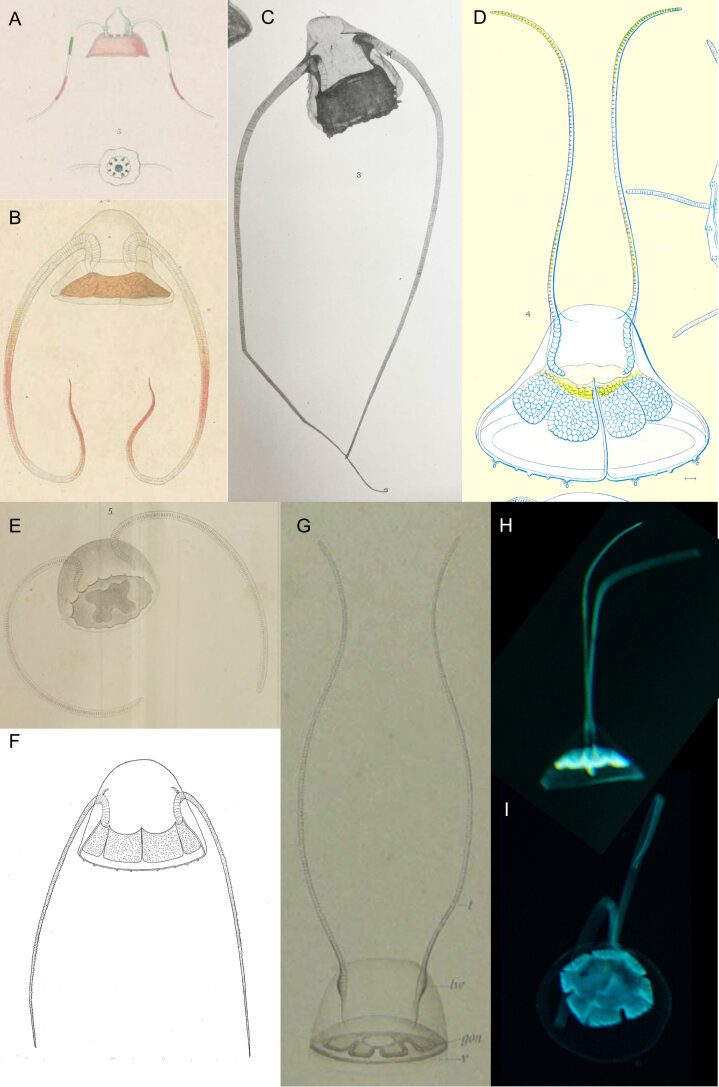
*Solmundellabitentaculata*. **A.** Drawing from the original description as *Carybdeabitentaculata* from Indonesia (Quoy and Gaimard 1833); **B.** Drawing of another specimen from Indonesia (Maas 1905); **C.** Photographed specimen from Eastern Pacific (bell diameter 3.5 mm) (Bigelow 1909); **D.** Drawing of a female specimen as *Solmundellamediterranea* from Florida (Mayer 1910); **E.** Drawing of a specimen as *Aeginopsismediterranea* from the Mediterranean Sea (Müller 1851); **F.** Drawing of a specimen from Japan (height 8.5 mm) (Uchida 1928); **G.** Drawing of a specimen as *Solmundellahenseni* from Florida (Maas 1893); **H-I.** Specimen MCMEC2018_Solmundella_bitentaculata_a observed on 27/11/2018: lateral view (H) and oral-lateral view (I). I-J photos courtesy: E. Cimoli.

Original description afterQuoy and Gaimard (1833)(basionym *Carybdeabitentaculata* Quoy & Gaimard, 1833) (Fig. 4A): umbrella consisting of two parts, with a heart-like, marquee-shaped upper part and a more flared, undulated (i.e. “limbe” in the original French version), lower part; two thin, long, rigid tentacles, with inside looking hollow, bending like horns and leaving from the junction between the two umbrella parts, penetrating deep inside the umbrella; large stomach, located deep in the umbrella, harbouring eight manubrial pouches; colour of the bottom of the medusa white or a red-gold yellow; colour of the tentacles reddish at the tip, greenish in the middle. Type locality: Ambon Bay, Indonesia.

Additional information from specimens from the Southern Ocean: There is currently only one species of *Solmundella*, though historically they were long dissociated into the species *S.bitentaculata* (Quoy & Gaimard, 1833) and *S.mediterranea* (Müller, 1851), which were subsequently synonymised (Kramp 1955, Thiel 1936, Kramp 1961). From **McMurdo Sound** (Browne 1910), reported as “*S.mediterranea*”, umbrella (up to seven mm wide) little broader than high, with a rather flat top, about on the level of the exit of the tentacles. Many small clusters of ectodermal cells scattered over the ex-umbrella, especially noticeable near the margin of the umbrella, containing many well-defined granules and generally harboured amongst those cells are a number of nematocysts. Four peronial grooves in the wall of the umbrella, cutting deep into the jelly at the margin of the umbrella, but of variable length and depth, with very conspicuous rudimentary grooves in each of the perradii without tentacles. The peronial band in each of the perradii without tentacles, runs alongside the sub-umbrella and turns off at the level of the stomach to the ex-umbrella, where there is a small funnel-shaped pit, showing a fair amount of variation. Gonads confined to the pouches of the stomach, but can extend over the lower part of to the stomach, nearly up to the mouth. Mouth circular. Tentacles 4-7 times as long as the umbrella diameter, of max. 40 mm in length. Margin of the umbrella invariably curled up. Up to eight sensory organs. Four minute interradial bulbs on the margin; from **Gauss Station** as *S.bitentaculata*, up to nine mm in diameter, one sensory organ (i.e. “Sinneshöcker” in the German original version) per quadrant, flanked by two or three rhopali [sic] (Vanhöffen 1912) (DL comment: probably a mis-interpretation and there was actually one tentacle bulb per quadrant, flanked by two or three statocysts).

Additional information from specimens from outside the Southern Ocean:*Solmundellabitentaculata* is a cosmopolitan species, which may actually be composed of multiple cryptic species (Lindsay et al. 2017). We, therefore, only give here a non-exhaustive list of descriptions of specimens from localities outside the Southern Ocean: in the **Mediterranean Sea** as *Aeginopsismediterranea* (Müller 1851) (Fig. 4E) and *S.mediterranea* (Metchnikoff 1886, Haeckel 1879) and in the Adriatic Sea (Neppi and Stiasny 1913); **Atlantic Ocean**: Canary Islands as *Aeginellabitentaculata* (Haeckel 1879), West Africa (only size of specimens given) (Kramp 1959), Florida current as *Solmundellahenseni* (Maas 1893) (Fig. 4G), Tortugas, Florida (Mayer 1910) (Fig. 4D), in Straits of Florida (Bigelow 1918) and middle and Southern Atlantic (between the latitudes 12°N - 63°S and longitudes 68°W - 21°E) (Thiel 1936); **Pacific Ocean**: Indonesia (Maas 1905) (Fig. 4B), Sea of Okhotsk and East China Sea (size only) (Bigelow 1913), Yellow Sea (Ling 1937), Japan (Uchida 1928) (Fig. 4F), north-east Australia (size only) (Blackburn 1955, Kramp 1953), Chile (size only) (Kramp 1952) and Eastern Pacific (Bigelow 1909) (Fig. 4C); **Indian Ocean**: Chagos Archipelago and Seychelles as *S.mediterranea* (Browne 1916) and off Madras, India (Menon 1932).

Characteristics of the observed material differing with previous descriptions (Fig. 4G-I): N = 1 in 2018. The shape of the bell (height 2/3 of width) was similar to the original description (i.e. upper marquee-shaped part and lower flatter part) (Fig. 4A) and, therefore, also similar to the drawings of Maas (1905) (Fig. 4B, from Indonesia) and Mayer (1910) (Fig. 4D, Florida), but differed from the rounder bell shape drawn by Müller (1851) (Fig. 4E, Mediterranean Sea), by Uchida (1928) (Fig. 4F, Japan) and the photograph in Bigelow (1909) (Fig. 4C, Eastern Pacific). Length of tentacles ca. four times bell height. Our specimen had stomach pouches showing jagged edges, whereas the shape of the stomach pouches of all previously described *S.bitentaculata* and synonyms was either omitted (e.g. Menon 1932, Browne 1910, Quoy and Gaimard 1833) or represented with smooth edges (e.g. Uchida 1928, Mayer 1910). Although hard to discern, it seems there are four tentacle buds with two statocysts between each one, matching the description by Browne (1910). The ex-umbrella was comprised of a pointed apical portion and a flared bell rim. No yellow or red colouration was observable.

### Leptomedusae

#### Leptomedusa sp. A

B2020F4A-E1F8-5DB6-89F9-C0C0C30C7FA3

##### Materials

- **Type status:**Other material. **Occurrence:** individualID: MCMEC2019_Leptomedusa_sp_A_a; lifeStage: adult; associatedMedia: http://morphobank.org/permalink/?P3993; **Taxon:** kingdom: Animalia; phylum: Cnidaria; class: Hydrozoa; order: Leptomedusae; **Location:** continent: Antarctica; waterBody: McMurdo Sound; maximumDepthInMeters: 1; decimalLatitude: -77.637; decimalLongitude: 166.401; **Identification:** identifiedBy: Dhugal Lindsay; **Event:** samplingProtocol: Sony Alpha 7 III camera equipped with a FE 90mm F2.8 Macro G OSS lens; eventDate: 2019-11-14; **Record Level:** type: StillImage; language: en; rightsHolder: Emiliano Cimoli

##### Notes

**Figure 5.**
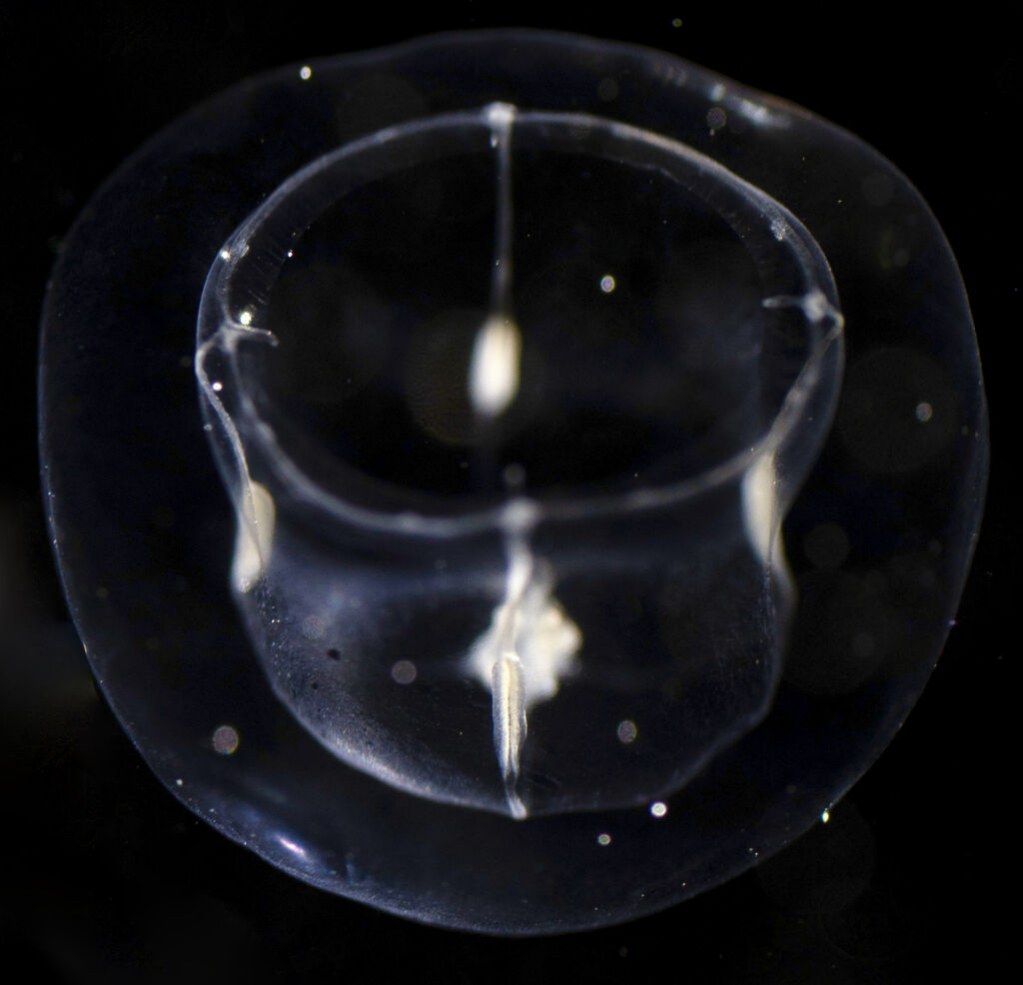
Leptomedusa species A (specimen MCMEC2019_Leptomedusa_sp_A_a) observed on 14/11/2019: oral-lateral view. Photo courtesy: E. Cimoli.

Description of and comments on observed material: N = 1 in 2019 (Fig. 5). Although it is hard to tell whether the medusa is bell-shaped or actually flattened but contracted, the “gonads” confined to the radial canals indicate that the observed specimen likely belongs to the order Leptothecata (or the accepted alternative synonym "Leptomedusae") (Bouillon et al. 2006). The presence of one manubrium excludes the family Sugiuridae and the gonads not extending on the manubrium excludes the family Tiarannidae. Four radial canals, gonads thin and located midway along the radial canals, four tentacle bulbs located perradially on the rim of the bell, one short tentacle per tentacle bulb, with nematocysts concentrated in tentacle's distal tip, medusa transparent, except for white-yellow gonads and manubrium. Diagnostic characters that could not been verified for further classification of the medusa: shape of manubrium, statocysts, cordyli, ocelli, gastric peduncle, tentacle bulbs and tentacular expansions. This medusa morphotype does not appear in the list of Larson and Harbison (1990) for medusae reported from the Ross Sea and we could not identify any other described species with the same morphological characteristics in the wider literature.

#### Leptomedusa sp. B

(Cosmetirella simplex sp. inc.)

F649419A-E6FF-5483-A401-8EAC31A7EAAD

##### Materials

- **Type status:**Other material. **Occurrence:** individualID: MCMEC2019_Leptomedusa_sp_B_a; lifeStage: adult; associatedMedia: https://youtu.be/hrufuPQ7F8U; **Taxon:** kingdom: Animalia; phylum: Cnidaria; class: Hydrozoa; order: Leptomedusae; **Location:** continent: Antarctica; waterBody: McMurdo Sound; maximumDepthInMeters: 1; decimalLatitude: -77.637; decimalLongitude: 166.401; **Identification:** identifiedBy: Dhugal Lindsay; **Event:** samplingProtocol: Sony Alpha 7 III camera equipped with a FE 90mm F2.8 Macro G OSS lens; eventDate: 2019-11-22; **Record Level:** type: StillImage, Video; language: en; rightsHolder: Emiliano Cimoli

##### Notes

**Figure 6.**
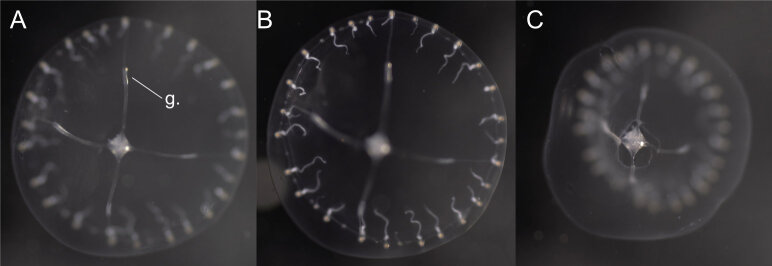
Leptomedusa species B (specimen MCMEC2019_Leptomedusa_sp_B_a) observed on 22/11/2019: oral view (A-B) and oral view when contracted (C). Abbreviation: g, gonad. Photos courtesy: E. Cimoli.

Description of and comments on observed material: N = 1 in 2019 (Fig. 6). This medusa showed the typical characteristics of the order Leptomedusae: flattened umbrella and gonads confined to the radial canals (Bouillon et al. 2006). The following families were excluded, based on the following observed diagnostic characters: only one manubrium (Sugiuridae), presence of statocysts or cordyli (Orchistomidae, Melicertidae and Dipleurosomatidae), gonads not extending on manubrium (Tiarannidae). Transparent medusa, four radial canals; 28 tentacles, short and white, with the inside of tentacle bulb light orange; sometimes one rudimentary tentacle bulb between each pair of tentacles; gonads very thin and linear located on the lateral canals, ca. one third of the radial canals away from the bell margin and length ca. one fifth of the length of the radial canal. Diagnostic characters that could not be definitively verified for further classification of the medusa: distinction and structure of statocyst or cordyli, gastric peduncle and manubrium connection to sub-umbrella. It resembles *Cosmetirellasimplex* Browne, 1910, which was described with 32 tentacles or more and which is currently regarded as synonymous with *Cosmetirelladavisii* (Browne, 1902) [basionym *Tiaropsisdavisii* Browne, 1902], which was described by Browne (1902) with about 80 tentacles around a 11 mm wide umbrella. When Kramp (1930) synonymised the species, he recognised a smaller growth form from the Antarctic with fewer (28-48) tentacles (i.e. *C.simplex*) and a larger form from the sub-Antarctic with more (56-150) tentacles (*C.davisii* + *C.kerguelensis*). Until these forms can be definitively assigned to the same species using molecular techniques, we tentatively assign the present individual to *C.simplex*, noting that, although cirri apparently are not present, we were unable to count the number or observe the type of statocysts for a definitive species identification.

### Siphonophorae

#### Pyrostephos vanhoeffeni

Moser, 1925

FE07A83C-DB7C-5162-8797-BC5A593B1624

##### Materials

- **Type status:**Other material. **Occurrence:** individualID: MCMEC2019_Pyrostephos_vanhoeffeni_a; lifeStage: adult; associatedMedia: "http://morphobank.org/permalink/?P3993", "https://youtu.be/R5E_HAW49DM", "https://youtu.be/o0XGpFavjyo", "https://youtu.be/QAADM0MERIo", "https://youtu.be/zEA6-7-qcYI", "https://youtu.be/SbuAA2nEVnU"; **Taxon:** scientificName: Pyrostephosvanhoeffeni; kingdom: Animalia; phylum: Cnidaria; class: Hydrozoa; order: Siphonophorae; family: Pyrostephidae; genus: Pyrostephos; **Location:** continent: Antarctica; waterBody: McMurdo Sound; maximumDepthInMeters: 1; decimalLatitude: -77.637; decimalLongitude: 166.401; **Identification:** identifiedBy: Dhugal Lindsay; **Event:** samplingProtocol: Sony Alpha 7 III camera equipped with a FE 90mm F2.8 Macro G OSS lens; eventDate: 2019-11-25; **Record Level:** type: StillImage, Video; language: en; rightsHolder: Emiliano Cimoli
- **Type status:**Other material. **Occurrence:** individualID: MCMEC2019_Pyrostephos_vanhoeffeni_b; lifeStage: adult; associatedMedia: "http://morphobank.org/permalink/?P3993", "https://youtu.be/tW2Ko92f3Bo", "https://youtu.be/2rrQCybEg0Q", "https://youtu.be/G9tev_gdUvQ", "https://youtu.be/NfJjKBRh5Hs", "https://youtu.be/1-aLzxLpzWs", "https://youtu.be/HnaIASH9yM0", "https://youtu.be/OSTJ3ldg63w", "https://youtu.be/d7OPyXn64g4", "https://youtu.be/YE50FZg8mpU", "https://youtu.be/csUoJl5Mapc", "https://youtu.be/uc6cP0YSrwc"; **Taxon:** scientificName: Pyrostephosvanhoeffeni; kingdom: Animalia; phylum: Cnidaria; class: Hydrozoa; order: Siphonophorae; family: Pyrostephidae; genus: Pyrostephos; **Location:** continent: Antarctica; waterBody: McMurdo Sound; maximumDepthInMeters: 1; decimalLatitude: -77.637; decimalLongitude: 166.401; **Identification:** identifiedBy: Dhugal Lindsay; **Event:** samplingProtocol: Sony Alpha 7 III camera equipped with a FE 90mm F2.8 Macro G OSS lens; eventDate: 2018-11-29; **Record Level:** type: StillImage, Video; language: en; rightsHolder: Emiliano Cimoli

##### Distribution

Antarctica: Ross Sea from under the ice (Totton 1965), from the Antarctic convergence to the south of the Bellingshausen and Weddell Seas (Mackintosh 1934), Croker Passage in the Antarctica Peninsula (Hopkins 1985, Panasiuk-Chodnicka et al. 2014, Panasiuk-Chodnicka and Żmijewska 2010), Drake Passage (Panasiuk et al. 2018), South Georgia Island (Alvariño 1981, Hardy and Gunther 1935), Weddell Sea (Pagès and Kurbjeweit 1994, Pagès and Schnack-Schiel 1996, Pagès et al. 1994, Panasiuk et al. 2018, Pugh et al. 1997), Falkland Islands (Alvariño 1981), Lützow-Holm Bay (Toda et al. 2010), Gauss Station (Moser 1925), D’Urville Sea (Grossmann 2010, Toda et al. 2014), Cosmonaut and Cooperation Seas (Margulis 1992), off the southern Victoria Land coast (71°2’S, 166°24’E) (Alvarino et al. 1990), east Antarctic (90°E) (Totton 1965); north of the Antarctic Convergence (54°6’S, 119°54’W) (Alvarino et al. 1990); Southern Chilean Fjords (Palma 2006); South Atlantic Ocean (Panasiuk et al. 2018), Argentina continental shelf (Araujo et al. 2013) and San Matias Gulf (Guerrero et al. 2013); New Zealand (Cairns et al. 2009).

##### Notes

**Figure 7.**
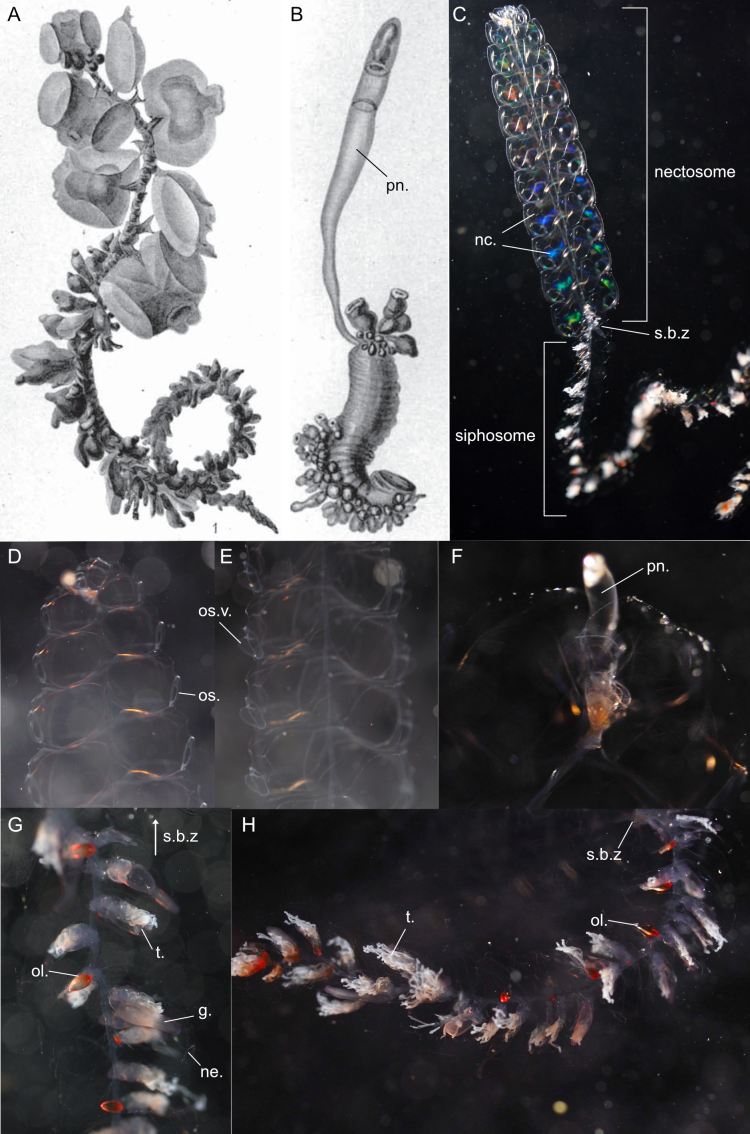
*Pyrostephosvanhoeffeni*. Drawing from the original account by Moser (1925) of a juvenile specimen (length ca. 25 mm) missing the pneumatophore and distal end (A) and a piece (length 6 mm) of another specimen including the nectosome with pneumatophore, but missing nectophores and a part of the siphosome (B), from Gauss Station, Antarctica. C-F. Specimen MCMEC2019_Pyrostephos_vanhoeffeni_b observed on 29/11/2019: whole specimen (C), apex nectosome (D), part of nectosome when contracting (E) and pneumatophore (F). G-H. Siphosome of specimen MCMEC2019_Pyrostephos_vanhoeffeni_a observed on 25/11/2019. Abbreviations: g., gastrozooids; nc., nectophore; ne., nematocysts; os., ostium; os. v., ostial velum; ol., oleocyst; pn., pneumatophore; s. b. z., siphosomal budding zone; t, tentacles. C-H photos courtesy: E. Cimoli.

Original description afterMoser (1925)(Fig. 7A-B): The original description is convoluted with much misapplied terminology, which was clarified and represented by Totton (1965), whose description we present below. Syntypes locality: Gauss Station (66.03°S, 89.63°E), Antarctica.

Description afterTotton (1965)of specimens at longitude 90°E, just off the Antarctic continent and from the Ross Sea from under the ice: we updated the terminology to describe Siphonophorae as in Pugh (2019). **Pneumatophore**: apex not pigmented. **Nectosome**: relatively long, two rows of nectophores. **Nectophores**: minimum 20 mm in length, carried on narrow muscular lamellae, which are inserted into a long bow-shaped mantle (i.e. “pallial”) canal that lies in a groove on the pedicular side (i.e. “adaxial side” in original description) of the nectophore. The pedicular canal (i.e. “adaxial canal”) bifurcates almost at once to form the upper (i.e. “dorsal”) and lower (i.e. “ventral”) radial canals. The two lateral radial canals arise separately from the upper canal and take an outward and ascending course on the pedicular nectosac-wall to cross over on to the lateral wall of the same. Here, each form first a small downward loop and then the main downward, lateral loop. After crossing under a fold in the lateral wall, each makes a final downward loop to run to the circular canal round the ostium. The lower radial canal is generally straight, but may have a few small bends. The upper canal usually has three or four more marked bends on the upper part of the nectosac. Nectosac has inpushing of the proximal (i.e. adaxial) side of its median part, absence of musculature from the median wall part similar to *Bargmannia* spp. or *Marrus* spp. **Siphosome**: dioecious (single-sexed), gonophores budding from one another to form small bunches, with male gonophores sausage shaped (size at maturity 1.3 × 0.5 mm) or female gonophores ovoid (0.5 mm in diameter), containing three to five eggs arranged meridionally and giving external appearance of the seams of a football. Continuous ventral line of budding gastrozooids (on the unsegmented stem), with young gastrozooids having an almost cylindrical basigaster and a conventional tentacle arising from a point very close to the junction of the basigaster with the pedicel (or pedicle). Mature gastrozooids (15 × 2 mm) spindle-shaped, consisting of three sections: the basigaster, the main stomach and a proboscis. Endodermal wall of the stomach vacuolated and consisting of four main types of cells. Each vacuole (ca. 0.07 mm in diameter) surrounded by 4-5 smaller more irregular vacuolated cells and forming transparent patches visible through the stomach wall. Small darker conical-shaped secretory cells (smaller than the large vacuoles) present in endoderm, located at the intersection of several cell boundaries, with their hemispherical surfaces projecting into the lumen of the gastrozooid and carrying stiff cilia (ca. 0.01 mm in length), similar to those described in the palpons of *Apolemia* by Willem (1894). Tentilla, up to 50 per tentacle. Hypertrophy of axial canal develops thickened mesogloeal walls and appears to form the extensile part of the cnidobattery, which has not been noted in any other species. It probably acts as an extensor on activation of the mechanism when the proximal end of the cnidoband breaks away from the pedicel and the whole cnidoband turns end to end and is flung on to the prey. In an early growth stage, the axial canal of a tentillum runs uniformly from the tentacle axis to the tip of the straight and short terminal filament. From the proximal end of this terminal filament, a diverticular canal of the axial canal runs back towards the pedicel alongside the proximal part of the axial canal. The epidermis on the opposite side to the axial canal of this diverticular canal forms the cnidoband. As growth proceeds, the lumen of the diverticular canal that lies under the cnidoband, comes to exceed the diameter of the axial canal and forms the cavity of the saccus. Five to seven lateral ovoid nematocysts (0.28 × 0.04 mm), located at the base of the tentillum’s saccus (i.e. "cnidosac"), which do not enter the wall of the saccus through the pedicel until the terminal filament, still in its straight uncoiled condition, reaches almost 0.5 mm in length. These nematocysts contain a large central structure which is probably the shaft with spines. The bracts (after Moser 1925) up to 23 × 14 mm in size, very thick, flat underneath, convex above,and proximally coming to a long point much broadened distally and irregularly toothed. The bracteal canal is slender, drawn out and ends some distance from the distal end, the latter seems to be three pointed. In early growth stages, a shallow horizontal pocket is found on the upper side between the lateral pair of points. The palpons (i.e. oleocysts) seem to be arranged at the dorsal edges of the ventral tract of siphosomal appendages, as if to buoy up the stem. **Colour**: stem orange in adults, vermilion in juveniles; nectophores wine red in adults, pink in juveniles; ostia carmine; gastrozooids golden red with fiery red mouth; cnidosacs fiery red.

Description of and comments on observed material (Fig. 7C-H): N = 3 in 2018, N = 4 in 2019. **Pneumatophore**: non-retractable transparent tube (Fig. 7F). **Nectosome**: up to 12 pairs of nectophores (Fig. 7C), lacking axial wings. A colony was observed jetting backwards (https://youtu.be/YE50FZg8mpU, https://youtu.be/uc6cP0YSrwc) by angling the ostial velum of the nectophores to deflect extruded water anteriorally (Fig. 7E). A similar velum alteration during forward and reverse swimming was previously observed in the physonect *Nanomiabijuga* (Mackie 1964, Costello et al. 2015). Ostium colourless (Fig. 7D). **Siphosome** (Fig. 7G-H): gastrozooids elongated, transparent and pink, oleocysts spherical to fusiform, oleocyst cavity transparent and bright red, the colour red being the brightest at the stem end of the oleocyst. Distal ridges of the bracts appear to be lined with nematocysts. Tentacles either contracted between the bracts or hanging down the siphosome with numerous white tentilla per tentacle. The number of pairs of nectophores vs. the minimum number of cormidia were counted for four individuals: 3p/31, 10p/27, 8p/12 and 12p/45.

### Semaeostomeae

#### Diplulmaris antarctica

Maas, 1908

F78108EF-8A23-56DE-A942-D196AF641F85

##### Materials

- **Type status:**Other material. **Occurrence:** individualID: MCMEC2019_Diplulmaris_antarctica_a; lifeStage: adult; associatedMedia: "http://morphobank.org/permalink/?P3993", "https://youtu.be/qKnd53wZVZo"; **Taxon:** scientificName: Diplulmarisantarctica; kingdom: Animalia; phylum: Cnidaria; class: Hydrozoa; order: Semaeostomeae; family: Ulmaridae; genus: Diplulmaris; **Location:** continent: Antarctica; waterBody: McMurdo Sound; maximumDepthInMeters: 1; decimalLatitude: -77.637; decimalLongitude: 166.401; **Identification:** identifiedBy: Dhugal Lindsay; **Event:** samplingProtocol: Sony Alpha 7 III camera equipped with a FE 90mm F2.8 Macro G OSS lens; eventDate: 2019-11-16; **Record Level:** type: StillImage, Video; language: en; rightsHolder: Emiliano Cimoli
- **Type status:**Other material. **Occurrence:** individualID: MCMEC2019_Diplulmaris_antarctica_b; lifeStage: juvenile; associatedMedia: "https://youtu.be/q9pcie-ri9M", "https://youtu.be/33EccdfSTh8", "https://youtu.be/kki0KyhFdUc"; **Taxon:** scientificName: Diplulmarisantarctica; kingdom: Animalia; phylum: Cnidaria; class: Hydrozoa; order: Semaeostomeae; family: Ulmaridae; genus: Diplulmaris; **Location:** continent: Antarctica; waterBody: McMurdo Sound; maximumDepthInMeters: 1; decimalLatitude: -77.637; decimalLongitude: 166.401; **Identification:** identifiedBy: Dhugal Lindsay; **Event:** samplingProtocol: Sony Alpha 7 III camera equipped with a FE 90mm F2.8 Macro G OSS lens; eventDate: 2019-11-30; **Record Level:** type: StillImage; language: en; rightsHolder: Emiliano Cimoli
- **Type status:**Other material. **Occurrence:** individualID: MCMEC2019_Diplulmaris_antarctica_c; lifeStage: juvenile; associatedMedia: "http://morphobank.org/permalink/?P3993", "https://youtu.be/pLlGoqwDZMs", "https://youtu.be/4PbHRjs4JVQ", "https://youtu.be/fh1rmQ_piZ8", "https://youtu.be/9MZ2BrZBLvE", "https://youtu.be/ce7Rvhf_8rw", "https://youtu.be/4XyQIQw04vs", "https://youtu.be/qDyH3_mnVBs", "https://youtu.be/NYDEDKs8PR0", "https://youtu.be/6EMBHjnJ7cU"; **Taxon:** scientificName: Diplulmarisantarctica; kingdom: Animalia; phylum: Cnidaria; class: Hydrozoa; order: Semaeostomeae; family: Ulmaridae; genus: Diplulmaris; **Location:** continent: Antarctica; waterBody: McMurdo Sound; maximumDepthInMeters: 1; decimalLatitude: -77.637; decimalLongitude: 166.401; **Identification:** identifiedBy: Dhugal Lindsay; **Event:** samplingProtocol: Sony Alpha 7 III camera equipped with a FE 90mm F2.8 Macro G OSS lens; eventDate: 2019-12-01; **Record Level:** type: StillImage, Video; language: en; rightsHolder: Emiliano Cimoli

##### Distribution

Southern Ocean: off Anvers Island, Antarctic Peninsula (Maas 1908), Ross Sea (USNM 53827, 58897) (Larson 1986, Browne 1910) and north of Ross Sea (62.408°S, 159.608°W, USNM 58895), Bellingshausen Sea (Larson 1986), Davis Sea (Larson 1986), off Dumont d’Urville (Thiebot et al. 2016, Toda et al. 2014), Gauss Station (as *Ulmaropsisdrygalskii*, Vanhöffen 1908), off cape Adare (Browne 1910) and in Prydz Bay (Australian Antarctic Data Centre 2018a, Hosie 1999b, Hosie 1999a). Although also reported from Madagascar (Richmond 1997) and the Indo-Pacific (van der Land 2008), these records are deemed unreliable and were probably misidentifications of *Diplulmarismalayensis* Stiasny, 1935.

##### Notes

**Figure 8.**
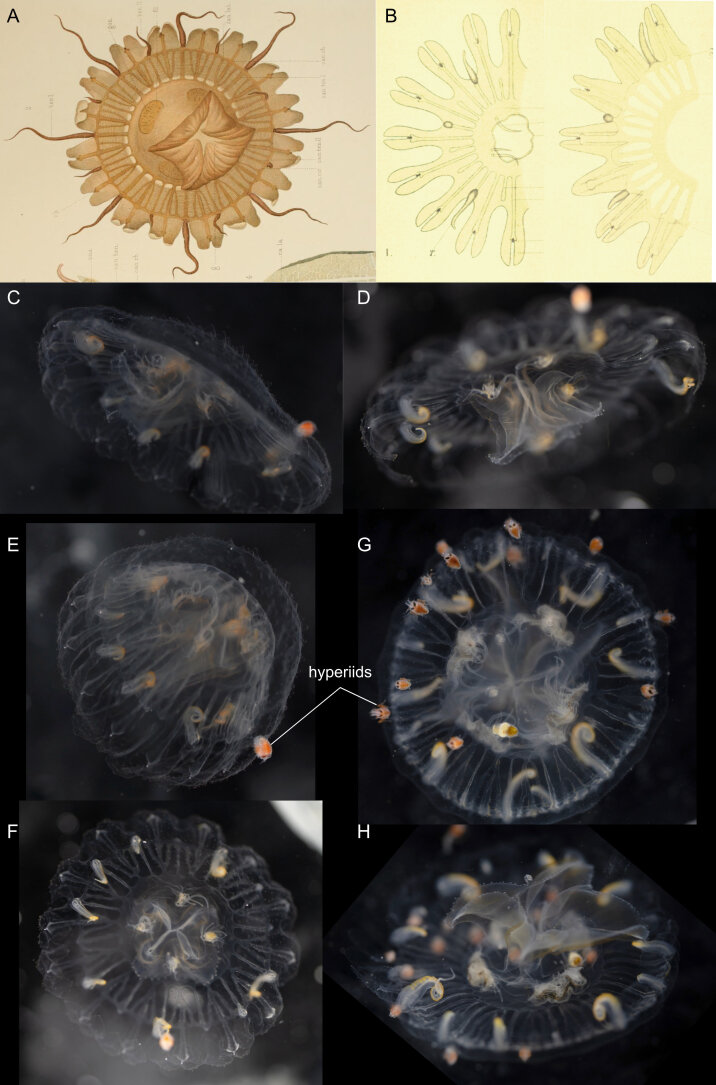
Juvenile *Diplulmarisantarctica*. **A.** Drawing from the original description (modified from Maas 1908) of a juvenile specimen (diameter 15 mm), oral view, from off Anvers Island, Antarctica; **B.** Drawing of a specimen in an early (left) and more advanced (right) ephyra stage from the Ross Sea, Antarctica (modified from Browne 1910); **C-E.** Juvenile specimen MCMEC2019_Diplulmaris_antarctica_b observed on 30/11/2019: apico-lateral view (C), lateral view (D), apico-lateral view when contracted (E) and apical view (F). G-H Juvenile specimen MCMEC2019_Diplulmaris_antarctica_c observed on 1/12/2019: apical view (G) and oral-lateral view (H). C-H photos courtesy: E. Cimoli.

**Figure 9.**
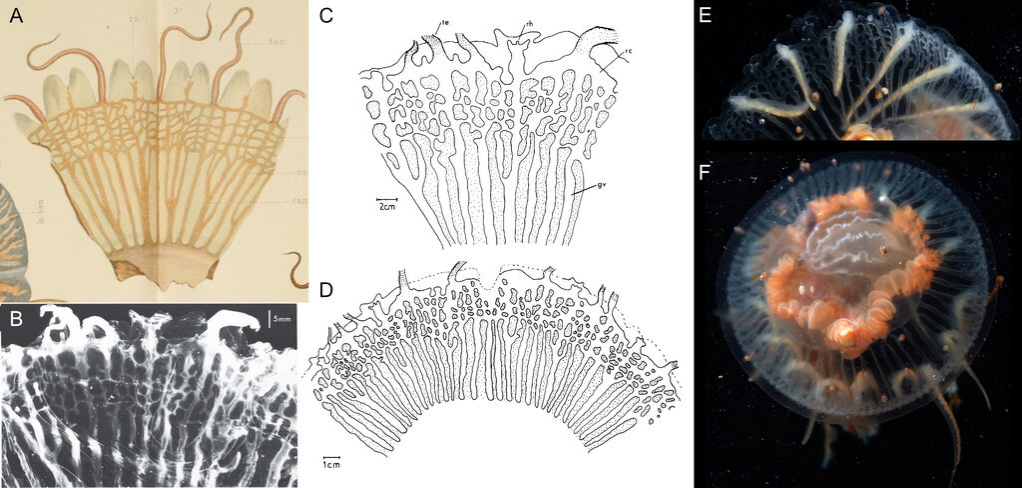
Adult *Diplulmarisantarctica*. **A.** Drawing from the original description by Maas (1908) of a bell portion of an adult specimen (diameter 35-40 mm), oral view, from off Anvers Island, Antarctica; **B.** Photography of sub-umbrella view of the gastrovascular canals of a specimen (diameter 18 cm) from McMurdo Sound (Operation Deep Freeze II station 61-D) (Larson 1986); **C-D.** Drawing of the sub-umbrella view of a specimen (diameter 10 cm) from McMurdo Sound (Operation Deep Freeze II station 61-D) (Larson 1986); **E-F.** Sub-umbrella (E) and apicular (F) views of an adult specimen MCMEC2019_Diplulmaris_antarctica_a observed on 16/11/2019 eating a *Beroe* sp. Gv: gastrovascular canal, rc: ring canal, rh: rhopalium, te: tentacle. E-F photos courtesy: E. Cimoli.

Original description afterMaas (1908)(Fig. 8A,Fig. 9A): medusa with 16 rhopalia, 16 tentacles and 32 marginal lappets, regularly alternating, with narrow canals, ramified at the periphery, connected through a circular canal. *Early stage* (15 mm diameter) (Fig. 8A): short manubrium, with quadrangular basal part more developed than its lips, the latter with little incisions, interradial arcs carrying the gastric filaments highly visible, also indicating the radius of the gonads, recognisable by a notch of the sub-umbrella with ectoderm and endoderm. Gastro-vascular system not consisting of pockets, but of real canals, due to the width of the merging anastomoses (i.e. “cathamnes” in the original French version). Two types of canals extend from the coronal base of the stomach (i.e. “basigaster coronaire”), distinctively separated at their origin: the canals in the radius of the 16 rhopalia and those in the radius of the tentacles, the former being separated close to their origins by anastomoses into a larger principal radial canal and into two lateral canals. The canals in the radius of the tentacles are divided into eight larger canals, similar to the canals in the axis of the rhopalia, with these eight canals being narrower in the axis of the less-developed tentacles compared to those in the axis of the largest tentacles. All those radial canals are reunited by a narrow continuous circular canal, which does not extend into the lappets themselves. The 16 statocysts are typically club-shaped and regularly placed. Of the 16 tentacles, eight are large and of equal size, the other eight are smaller and of inequal size. The bell margin is incised into 16 large primary lappets at the radius of the tentacles, with these incisions being the deepest in the radius of the eight large tentacles, whereas the eight other incisions at the smaller tentacles are shallower and more unequal, with some barely incised at all. The 16 incisions in the radius of the statocysts are shallower, but of equal depth, dividing each primary lappet into two secondary ones. *Adult stage* (35-40 mm diameter, description based on one quadrant) (Fig. 9A): the incisions are more or less equal and the difference in size amongst tentacles is less marked. The disc grew mainly in the area located between the stomach and the periphery of the lappets. The canals were elongated and became single lanes of communication between the central sinus and the peripherical network, the latter being formed due to the increased complexity of the canal branches without significant growth, the mesh becoming, therefore, more irregular. Gonads more distinct. Type locality: Antarctica, off Anvers Island (during the French Antarctic Expedition with the "*Français* " vessel).

Additional information from specimens from the Southern Ocean: from **Gauss Station** as *Ulmaropsisdrygalskii* (Vanhöffen 1908), description matching with the original of Maas (1908), 96 bases of canals leaving the stomach. *Meta-ephyra stage*: for medusa of 15 mm diameter, the beginning of the formation of lateral canals emerging from the canal in the axis of the rhopalia can be observed, at 17 mm diameter, these lateral canals are more defined and, by 22 mm diameter, two pairs of lateral canals are present, although the anastomoses for these canals are still missing; from the **Ross Sea** (Browne 1910) (Fig. 8B), *ephyra stage* (smallest 4-5 mm in diameter), with 16 fairly long arms divided into two flat lobes, 32 straight unbranched radial canals, 16 of which directly run from the stomach to the rhopalia and alternating with 16 in the axis of the tentacles, the latter developed slightly later than the rhopalial canals. Tentacles in rudimentary stage, either as bulb-like buds, tapering elongated buds or minute tentacles. In the smallest ephyra, only four of those tentacular buds present and an additional 12 buds develop, in irregular intervals and without any definite order, as the medusa grows. Stomach small and circular, with four gastric filaments (number increasing as medusa grows), with one filament in each group much longer than the others in the early developmental stages. Mouth simple large opening, without any definite lips or arms, which appear later. Ex-umbrella covered with small clusters of nematocysts, which, in later stages, will be confined to the aboral side of the marginal lobes. Circular canal formed by outgrowths from the radial canals and formed before the branches of the rhopaliar canals begin to develop. *Meta-ephyra stage* (15-25 mm diameter) similar to Maas (1908). *Adult stage* (three specimens of diameter between 60-75 mm, none of which were complete), umbrella thin, margin of the mouth studded with warts and short protuberances containing nematocysts, stomach is a flat circular cavity (size 2/3 of umbrella diameter), covered in a moderately thick layer of mesoglea on its lower side. Radial canals as described by Maas (1908). Gonads narrow band on the outer side of the gastric filaments, protruding from the stomach and hanging down from the sub-umbrella and becoming broader and sinuously folded when further developed. Tentacles hollow and laterally compressed, especially in their basal portions, but the distal portion is rounder and tapers off to a slender tip. Along the whole inner side of the tentacle runs a band, closely studded with nematocyst clusters. In fully-grown tentacles, the inner cavity has transverse folds. Number of rhopalia and tentacles same as Maas (1908). Rhopalia not well-protected, situated on the wall of the niche formed by the marginal lobes and pointing upwards to the aboral side of the umbrella. Rhopaliar canal, leading from the circular canal to the sense organs, broad and flat. Over the rhopaliar canal and on the surface of the umbrella, a small patch of darkly-coloured cells is present, forming a rudimentary dorsal sensory pit, which is occasionally absent. Ex-umbrella side of marginal lobes covered in numerous warts containing nematocysts. Lobes show slight variation in shape and fill up the space between the sense organs and the tentacles; from the **Bellingshausen Sea**, **Ross Sea** and **Davis Sea** (Larson 1986) (Fig. 9B-D), adult stage (bell diameters 90-180 mm), umbrella mesoglea thin, ex-umbrella smooth, rounded or pointed marginal lappets, 16 rhopalia alternating with 1-3 marginal tentacles. Tentacles (number between 16 and 48), laterally compressed, with abaxial nematocyst warts along their length. Four oral arms, frilled, curtain-like, length ca. equal to bell diameter, lip margin studded with nematocyst papillae. Four gonads, everted and sac-like. Gastrovascular canals between 32-96. Rhopaliar canals 16, with 1-5 interjacent tentacular canals between each rhopalium. All canals proximally unbranched for inner 1/2- 2/3 of length, distally anastomosing in an irregular network. Ring canal near umbrella margin. Colour: umbrella colourless, tentacles whitish, gastrodermis of stomach and of oral arms reddish-orange.

Additional information from specimens from outside the Southern Ocean: to our knowledge, no specimens have been described outside the Southern Ocean. The records from Madagascar (Richmond 1997) and the Indo-Pacific (van der Land 2008), are not accompanied by photographs or morphological descriptions and, although the sketch/illustration of "*Diplulmarisantarctica*" in Richmond (1997) shows the characters of the genus, both records are deemed mis-identifcations of *Diplulmarismalayensis* Stiasny, 1935, or another presently undescribed congener. Expatriation of Southern Ocean species is not unknown, so further surveys in southern Africa, Chile or New Zealand should carefully investigate the species-specific characters.

Comments on observed material: N = 3 in 2019 [two juveniles (Fig. 8G-H) and one adult specimen (Fig. 9E-F), based on the ramification of peripheral canals]. Bell diameter of one juvenile specimen, ca. 50 mm; ex-umbrella covered in warts and cnidocysts, these warts being large and pointy in young medusa and smaller and rounder in the adult specimen; dorsal surface of marginal lappets covered in cnidocysts; transparent tubular gastric filaments rooted at the four corners of the manubrium; gastrodermis of adult orange; oral arms transparent, length ca. same as bell radius, with frilled edges; tentacles white with yellow segmented dorsal side; hyperiids attached to ex-umbrella; small transparent *Beroe* sp. in stomach of adult specimen.

### Beroida

#### Beroe

Müller, 1776

F2584E72-9205-527E-8082-9BBB9CC1952D

##### Notes

*Description of the genus Beroe*: sac-like bodies without tentacles or tentacle sheaths, very large mouth and stomodaeum, eight meridional canals, connected orally and a row of branched papillae in a figure of eight at the aboral pole (Licandro and Lindsay 2017). The identification of *Beroe* species can be very difficult, as of the current 27 described species (WoRMS Editorial Board 2020), many have only been superficially described and the number of synonyms is likely very high (Harbison et al. 1978). We, therefore, only report the morphological characteristics of the three *Beroe* species reported for the Southern Ocean (Table 1), namely: *Beroecompacta* Moser, 1909 from Gauss Station (e.g. Moser 1909) (Fig. 10D) and Eastern Antarctica (e.g. Grossmann 2010), "*Beroecucumis*" from the Antarctic Peninsula (e.g. Friedlander et al. 2020) and Gauss Station (e.g. Moser 1909) and "*B.ovale*" in the northern Ross Sea (e.g. Ocean Survey 20/20 2013). O'Sullivan (1986) also mentioned that *B.forskalii* was recorded from the Antarctic Peninsula in Chun (1880), within his monograph on ctenophores from the Gulf of Naples and surrounding waters, but we were unable to find such a geographical record in that manuscript. A photograph of a 64 mm-long Beroe species from the Danco coast of the Antarctic Peninsula (64.65˚S, 61.916˚W) appears in Figure 3 of Whelan et al. (2017). It is whitish, but appears to have yellowish-brown pigment around the mouth and in the distal halves of the meridional canals, with the diverticula not anastomosing. Only a single photograph is presented, without notes on morphology, but the nuclear 18S ribosomal RNA sequence (MF599315) should allow subsequent authors to characterise it. In any case, the comb rows are too short and too long, respectively, for it to be assignable to the present *Beroe* sp. A or B. Regarding "*B.ovale*" (Fig. 10a), apparently the Match Taxa tool in the World Register of Marine Species (WoRMS Editorial Board 2020) at that time was used to finalise the species assignment, but the rationale for identification has not been recorded. We consider it most likely that the recorded species resembled either *B.ovata* sensu Mayer, 1912 (Fig. 10c) = *B.ovata* Chamisso & Eysenhardt, 1821 = *B.ovatus* Bosc, 1802 or *B.ovata* sensu Chun, 1880 (Fig. 10b) = *B.ovata* Eschscholtz, 1829 = *B.ovata* Bruguière, 1789 [two different species - see Mills et al. 1996]. The WoRMS taxonomy for *B.ovale* Bosc, 1802 has been updated to the correct species epiphet (i.e. *B.ovatus* Bosc, 1802).

#### Beroe sp. A

965476A7-D140-5D43-95F8-B9EDF3DFD6AC

##### Materials

- **Type status:**Other material. **Occurrence:** individualID: MCMEC2019_Beroe_sp_A_a; lifeStage: adult; associatedMedia: http://morphobank.org/permalink/?P3993; **Taxon:** kingdom: Animalia; phylum: Ctenophora; class: Nuda; order: Beroida; genus: Beroe; **Location:** continent: Antarctica; waterBody: McMurdo Sound; maximumDepthInMeters: 1; decimalLatitude: -77.637; decimalLongitude: 166.401; **Identification:** identifiedBy: Dhugal Lindsay; **Event:** samplingProtocol: Sony Alpha 7 III camera equipped with a FE 90mm F2.8 Macro G OSS lens; eventDate: 2019-11-16; **Record Level:** type: StillImage, Video; language: en; rightsHolder: Emiliano Cimoli
- **Type status:**Other material. **Occurrence:** individualID: MCMEC2019_Beroe_sp_A_b; lifeStage: juvenile; associatedMedia: "https://youtu.be/kGBUQ7ZtH9U", "https://youtu.be/Vbl_KEmPNmU"; **Taxon:** kingdom: Animalia; phylum: Ctenophora; class: Nuda; order: Beroida; genus: Beroe; **Location:** continent: Antarctica; waterBody: McMurdo Sound; maximumDepthInMeters: 1; decimalLatitude: -77.637; decimalLongitude: 166.401; **Identification:** identifiedBy: Dhugal Lindsay; **Event:** samplingProtocol: Sony Alpha 7 III camera equipped with a FE 90mm F2.8 Macro G OSS lens; eventDate: 2019-11-30; **Record Level:** type: Video; language: en; rightsHolder: Emiliano Cimoli
- **Type status:**Other material. **Occurrence:** individualID: MCMEC2018_Beroe_sp_A_c; lifeStage: adult; associatedMedia: "http://morphobank.org/permalink/?P3993", "https://youtu.be/trWsOI6g-9Y"; **Taxon:** kingdom: Animalia; phylum: Ctenophora; class: Nuda; order: Beroida; genus: Beroe; **Location:** continent: Antarctica; waterBody: McMurdo Sound; maximumDepthInMeters: 1; decimalLatitude: -77.637; decimalLongitude: 166.401; **Identification:** identifiedBy: Dhugal Lindsay; **Event:** samplingProtocol: Sony Alpha 7 III camera equipped with a FE 90mm F2.8 Macro G OSS lens; eventDate: 2018-11-16; **Record Level:** type: StillImage; language: en; rightsHolder: Emiliano Cimoli
- **Type status:**Other material. **Occurrence:** individualID: MCMEC2018_Beroe_sp_A_d; lifeStage: juvenile; associatedMedia: http://morphobank.org/permalink/?P3993; **Taxon:** kingdom: Animalia; phylum: Ctenophora; class: Nuda; order: Beroida; genus: Beroe; **Location:** continent: Antarctica; waterBody: McMurdo Sound; maximumDepthInMeters: 1; decimalLatitude: -77.637; decimalLongitude: 166.401; **Identification:** identifiedBy: Dhugal Lindsay; **Event:** samplingProtocol: Sony Alpha 7 III camera equipped with a FE 90mm F2.8 Macro G OSS lens; eventDate: 2018-11-25; **Record Level:** type: StillImage; language: en; rightsHolder: Emiliano Cimoli
- **Type status:**Other material. **Occurrence:** individualID: MCMEC2018_Beroe_sp_A_e; lifeStage: juvenile; associatedMedia: http://morphobank.org/permalink/?P3993; **Taxon:** kingdom: Animalia; phylum: Ctenophora; class: Nuda; order: Beroida; genus: Beroe; **Location:** continent: Antarctica; waterBody: McMurdo Sound; maximumDepthInMeters: 1; decimalLatitude: -77.637; decimalLongitude: 166.401; **Identification:** identifiedBy: Dhugal Lindsay; **Event:** samplingProtocol: Sony Alpha 7 III camera equipped with a FE 90mm F2.8 Macro G OSS lens; eventDate: 2018-11-27; **Record Level:** type: StillImage; language: en; rightsHolder: Emiliano Cimoli
- **Type status:**Other material. **Occurrence:** individualID: MCMEC2019_Beroe_sp_A_f; lifeStage: adult; associatedMedia: http://morphobank.org/permalink/?P3993; **Taxon:** kingdom: Animalia; phylum: Ctenophora; class: Nuda; order: Beroida; genus: Beroe; **Location:** continent: Antarctica; waterBody: McMurdo Sound; maximumDepthInMeters: 1; decimalLatitude: -77.637; decimalLongitude: 166.401; **Identification:** identifiedBy: Dhugal Lindsay; **Event:** samplingProtocol: Sony Alpha 7 III camera equipped with a FE 90mm F2.8 Macro G OSS lens; eventDate: 2018-11-17; **Record Level:** type: StillImage; language: en; rightsHolder: Emiliano Cimoli
- **Type status:**Other material. **Occurrence:** individualID: LRISH2010_Beroe_sp_A_g; lifeStage: adult; associatedMedia: http://morphobank.org/permalink/?P3993; **Taxon:** kingdom: Animalia; phylum: Ctenophora; class: Nuda; order: Beroida; genus: Beroe; **Location:** continent: Antarctica; waterBody: Little Razorback Island; **Identification:** identifiedBy: Gerlien Verhaegen; **Event:** eventDate: 2010-12-02; **Record Level:** type: StillImage; language: en; rightsHolder: Shawn Harper

##### Distribution

A similar brownish-orange undescribed *Beroe* species has been observed in Antarctica, in the Ross Sea (Brueggeman 1998) (Fig. 11d) and in the Weddell Sea (photographed by ©Ingo Arndt, https://www.mindenpictures.com/search?s=Ingo+Arndt+beroe). Another pink-orange *Beroe* was collected and photographed off Argentina, but the specimen was too damaged to properly identify (see Figure 2F in Schiariti et al. 2021).

##### Notes

Description of and comments on observed material (Fig. 11): N = 3 in 2018 and N = 4 in 2019. Body shape long and oval (body length ca. 2.4 times body width), eight comb rows above the meridional canals, starting close to the aboral end, of equal length ca. 2/3 of the body length [ca. 85 comb plates per row in Little Razorback Island LRISH2010_Beroe_sp_A_g specimen], meridional canals extend past the oral end of the comb rows, space between comb plates short (space between four comb plates ca. equal to length of comb plate); white and brownish-orange divertula without anastomoses; orange-brown stomodeum, its length nearly full body length. None of the currently described *Beroe* species found in the Southern Ocean matched with the description of our specimens (Table 1). Although colour supposedly is of little taxonomic significance to distinguish between *Beroe* species (e.g. Arai 1988, Quoy and Gaimard 1824), it is the first time that brownish-orange specimens have been formally described for this genus in the Southern Ocean (Table 1). Observed swimming or immobile at any depths between the seafloor and the surface ice cover, orientated either horizontally or vertically (with aboral end pointing up or down).

#### Beroe sp. B

93ACA6B5-891E-52D9-98FF-CD19AD5D2D4D

##### Materials

- **Type status:**Other material. **Occurrence:** individualID: MCMEC2019_Beroe_sp_B_a; associatedMedia: "http://morphobank.org/permalink/?P3993", "https://youtu.be/VC-peoIaI0I"; **Taxon:** kingdom: Animalia; phylum: Ctenophora; class: Nuda; order: Beroida; genus: Beroe; **Location:** continent: Antarctica; waterBody: McMurdo Sound; maximumDepthInMeters: 1; decimalLatitude: -77.637; decimalLongitude: 166.401; **Identification:** identifiedBy: Dhugal Lindsay; **Event:** samplingProtocol: Sony Alpha 7 III camera equipped with a FE 90mm F2.8 Macro G OSS lens; eventDate: 2019-11-15; **Record Level:** type: StillImage, Video; language: en; rightsHolder: Emiliano Cimoli

##### Distribution

First reported observation.

##### Notes

**Figure 12.**
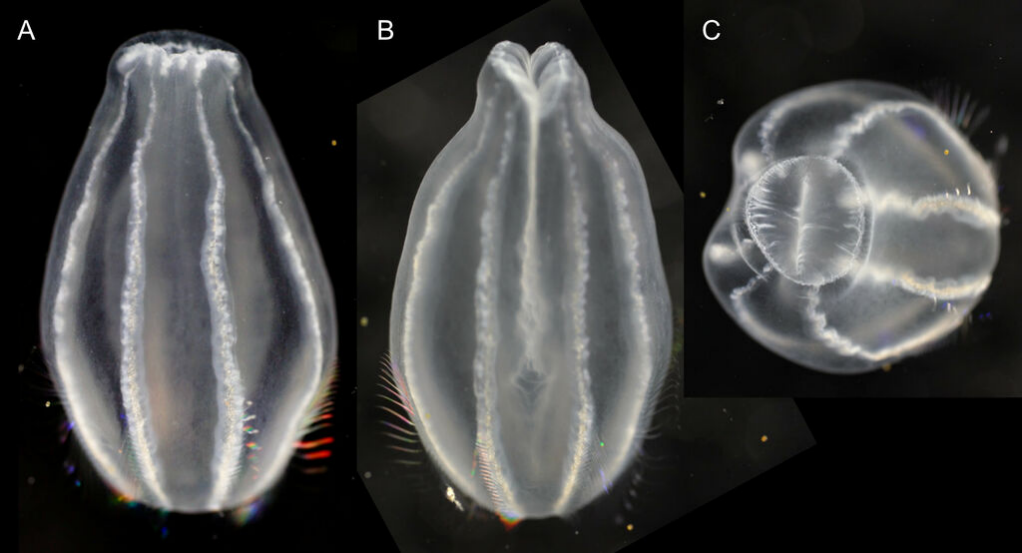
*Beroe* sp. B specimen MCMEC2019_Beroe_sp_B_a observed on 15/11/2019: lateral views (A-B) and oral view (C). Photos courtesy: E. Cimoli.

Description of and comments on observed material: N = 1 in 2019 (Fig. 12). Body length ca. 35 mm, body shape oval (body length ca. 1.5 times body width); colour transparent to milky white; length of comb rows 1/4 of the body length; ca. 22 comb plates per comb row; space between five comb plates ca. equal to width of comb plate and length of comb plates four times the width of a comb plate; length of meridional canals full body length; length stomodeum ca. equal to body length; no diverticula; small yellow round deposits located within the walls of the meridional canals (could be gonads, sperm, eggs or oil droplets). None of the currently described adult *Beroe* species found in the Southern Ocean matched with the description of our specimen (Table 1); however, the lack of colour and the shortness of the comb rows vs. body length could indicate our specimen was a juvenile. Indeed, despite the colour of juvenile individuals being rarely included in the description or re-description of *Beroe* species, when reported, juveniles are either colourless (e.g. *B.abyssicola* Mortensen, 1927), transparent (e.g. *B.ovata* *sensu* Chun, 1880 and *B.pandorina* Moser, 1903) or of lighter colour (e.g. *B.abyssicola* in Arai 1988) compared to intraspecific adults. Shorter comb rows vs. body length in larvae or young specimens compared to adults have been reported for *B.campana* (Komai 1918), *B.forskalii* (Moser 1903), *B.gracilis* (Künne 1939), *B.mitrata* (Moser 1907), *B.ovata* *sensu* Mayer, 1912 (Mayer 1912) and *B.ramosa* (Komai 1921). However, even when shorter, the comb length in juveniles of the *Beroe* species listed above usually extended up to half the body length. Therefore, the comb row length of our observed species is unusually short, regardless of its life stage and, therefore, likely it is a *Beroe* species not yet reported to occur in the Southern Ocean.

### Cydippida

#### Callianira cristata

Moser, 1909

87EF759E-4F79-5526-88EF-F414494C1E96

##### Materials

- **Type status:**Other material. **Occurrence:** individualID: MCMEC2019_Callianira_cristata_a; lifeStage: adult; associatedMedia: "https://youtu.be/30g9CvYh5JE"; **Taxon:** scientificName: Callianiracristata; kingdom: Animalia; phylum: Ctenophora; class: Tentaculata; order: Cydippida; family: Cydippida incertae sedis; genus: Callianira; **Location:** continent: Antarctica; waterBody: McMurdo Sound; maximumDepthInMeters: 1; decimalLatitude: -77.637; decimalLongitude: 166.401; **Identification:** identifiedBy: Dhugal Lindsay; **Event:** samplingProtocol: Sony Alpha 7 III camera equipped with a FE 90mm F2.8 Macro G OSS lens; eventDate: 2019-11-22; **Record Level:** type: Video; language: en; rightsHolder: Emiliano Cimoli
- **Type status:**Other material. **Occurrence:** individualID: MCMEC2019_Callianira_cristata_b; lifeStage: adult; associatedMedia: http://morphobank.org/permalink/?P3993; **Taxon:** scientificName: Callianiracristata; kingdom: Animalia; phylum: Ctenophora; class: Tentaculata; order: Cydippida; family: Cydippida incertae sedis; genus: Callianira; **Location:** continent: Antarctica; waterBody: McMurdo Sound; maximumDepthInMeters: 1; decimalLatitude: -77.637; decimalLongitude: 166.401; **Identification:** identifiedBy: Dhugal Lindsay; **Event:** samplingProtocol: Sony Alpha 7 III camera equipped with a FE 90mm F2.8 Macro G OSS lens; eventDate: 2019-11-20; **Record Level:** type: StillImage; language: en; rightsHolder: Emiliano Cimoli

##### Distribution

Antarctica: Ross Sea [photographed by ©Shawn Harper in Brueggeman (1998)], Gauss Station (Moser 1909), north of Prydz Bay (Australian Antarctic Data Centre 2019, Australian Antarctic Data Centre 2018b, Australian Antarctic Data Centre 2018c, Hosie 1991, Hosie 1999b) and west of Cape Adare (Crossley and Hoddell 2017).

##### Notes

**Figure 13.**
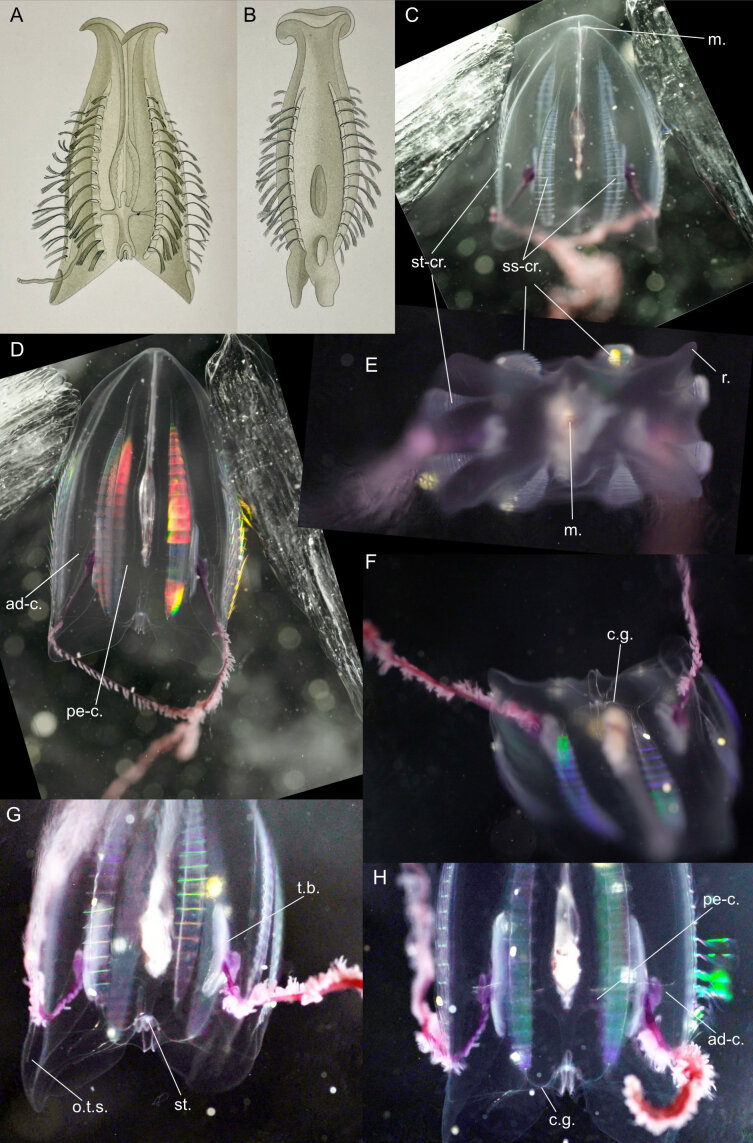
*Callianiracristata*. **A-B.** Drawing from the original description (specimen length 12 mm) (modified from Moser 1909) from Gauss Station, Antarctica: lateral view of the substomodeal plane (A) and the subtentacular plane (B); **C-H.** Specimen MCMEC2019_Callianira_cristata_a observed on 22/11/2018: lateral-substomodeal views (C-D, G-H) oral view (E) and latero-aboral view (F). The contrast and brightness of images G-H were strongly modified to reveal underlying morphological structures. Abbreviations: ad-c., adradial canal; c.g., ciliary groove; m., mouth; o.t.s., opening of tentacle sheath; pe-c., perradial canal; r., ridge; st., statocyst; ss-cr., substomodeal comb row; st-cr., subtentacular comb row; t.b., tentacle bulb. C-H photos courtesy: E. Cimoli.

Original description after Moser (1909) (Fig. 13A-B): length specimens 2-13 mm, body slim, slightly flattened at the stomodaeal region. Two keels at the aboral pole (i.e. “Sinnespol” in the German original version), the body gradually widening from the mouth onwards when viewed from the substomodeal plane, while appearing cylindrical and tapered towards both ends when viewed from the subtentacular plane. Lip-shaped protuberance (i.e. “Sinneskörper”) near statocyst missing. The keels are wide and short, similar to those of *Callianiraantarctica*, but much shorter compared to those of *Callianirabialata*. The oval opening of the tentacle sheath is located laterally directly under the blunt tips of the keels, lower compared to *C.antarctica*. The comb rows lie on strong protruding meridional ridges, between which the body surface is concave. The comb rows are very long, with the substomodeal comb rows running from the height of the statocyst to one-fifth of the body length from the oral end, whereas the shorter subtentacular comb rows run from slightly deeper in the base of the wings to one-quarter of the body length from the oral end. The comb plates (i.e. “Schwimmplättchen”) lie on strongly protruding basal swellings (i.e. “Basalwülsten”); they are narrow and very long, the longest comb plates found in the aboral third of the body, from where they shorten fairly quickly in length towards the aboral pole and gradually shorten towards the oral pole. The space between the comb plates of the substomodeal comb rows is wider compared to the subtentacular ones. Two long polar plates slide between the aboral ends of the substomodeal comb rows. Mouth small and is bound by two lips lying in the stomodaeal plane. Stomach very slim and long (length ca. 4/5 body length), with short swellings. Perradial canals (i.e. “Trichtergefäß”) short, rather thick pipes. Statocyst exposed. The adradial canals (i.e. “adradialen Gefäße”) enter the meridional canals (i.e. “Meridionalgefäße") at the same height as the infundibulum (i.e. “Trichter”). Tentacle bulbs short and wide, located very low, at the same height as the infundibulum, between the infundibulum and the body wall. The tentacle bulbs are pointed orally, split aborally into two short tips and have in their middle, both proximally and distally, each a short cone, from which the tentacle arises. Tentacle sheath (i.e. “Scheide”) short and very wide, with small oval openings near the tip of the keels. Colour not stated, likely colourless. Compared to *C.antarctica*, the only other *Callianira* species reported from the Southern Ocean (e.g. Kaufmann et al. 2011, Moser 1909, Scolardi et al. 2006, Sherlock et al. 2011), according to Moser (1909), *C.cristata* has narrower and extremely long comb plates and the tentacle sheath opening is closer to the tip of the keels and it has longer tentacle bulbs. Type locality: Gauss station (66.03˚S, 89.63˚E), Antarctica.

Description and comments of observed material: N = 1 in 2019 (Fig. 13C-H). The morphological **similarities** observed compared to the original description of Moser (1909) were the following: body shape, keel length vs. total body length (including keel) 17%, opening of tentacle sheath located at the tip of the keels, length of substomodeal comb rows longer than subtentacular comb rows (ca. 25 vs ca. 17), comb plates laying on protruding basal swellings, long comb plates with the longest found in aboral third of the body (length of subtentacular comb plates 2.5 times the inter-comb plate distance in aboral third of body), mouth shape, slim and long pharynx (ca. 65% of body length), short and thick perradial canals, adradial canals entering meridional canals at the height of infundibulum, tentacle bulbs split aborally in two, tentacle arising from a short cone located at the middle of the tentacle bulb, polar plate length up to the second comb plate from the aboral end of the substomodeal comb rows. **Dissimilarities**: raised ridges present near statocyst (Fig. 13); many more comb plates per comb row, with ca. 25 comb plates (Fig. 12D) for substomodeal comb rows vs. 14 (Fig. 13A) on the drawing of Moser (1909); body colourless, except for dark purple tentacle bulb and tentacles, with numerous short light purple tentilla. **New reported characteristics**: six ridges (Fig. 13E), one between each substomodeal comb row and subtentacular comb row and also between adjacent substomodeal comb rows; ciliary groove running from the aboral end of the substomodeal meridional canals to the aboral pole (Fig. 13H); in the oral third of the body, space between two substomodeal comb plates ca. equal to width of comb. It is worth noting that the validity of the genus *Callianira* is currently under debate and in need of a thorough revision, as the arguments to join its first three species did not meet modern standards (Bennema and van Moorsel 2011). A recent phylogenetic study of Ctenophora, including *Callianiraantarctica*, even suggested that *Callianira* should be excluded from the family Mertensiidae and should remain as *incertae familae* until further revision (Townsend et al. 2020).

#### "fam. Mertensiidae" sp. A

158D3806-104E-54C6-B263-0013992ECD3C

##### Materials

- **Type status:**Other material. **Occurrence:** individualID: MCMEC2019_Mertensiidae_sp_A_a; associatedMedia: "http://morphobank.org/permalink/?P3993", "https://youtu.be/dKELHUITnlg", "https://youtu.be/GE6WgN8VBdw", "https://youtu.be/0W2HHLW71Pw"; **Taxon:** kingdom: Animalia; phylum: Ctenophora; class: Tentaculata; order: Cydippida; family: Mertensiidae; **Location:** continent: Antarctica; waterBody: McMurdo Sound; maximumDepthInMeters: 1; decimalLatitude: -77.637; decimalLongitude: 166.401; **Identification:** identifiedBy: Dhugal Lindsay; **Event:** samplingProtocol: Sony Alpha 7 III camera equipped with a FE 90mm F2.8 Macro G OSS lens; eventDate: 2019-11-15; **Record Level:** type: StillImage, Video; language: en; rightsHolder: Emiliano Cimoli
- **Type status:**Other material. **Occurrence:** individualID: MCMEC2019_Mertensiidae_sp_A_b; associatedMedia: "https://youtu.be/pvXYlQGZIVg"; **Taxon:** kingdom: Animalia; phylum: Ctenophora; class: Tentaculata; order: Cydippida; family: Mertensiidae; **Location:** continent: Antarctica; waterBody: McMurdo Sound; maximumDepthInMeters: 1; decimalLatitude: -77.637; decimalLongitude: 166.401; **Identification:** identifiedBy: Dhugal Lindsay; **Event:** samplingProtocol: Sony Alpha 7 III camera equipped with a FE 90mm F2.8 Macro G OSS lens; eventDate: 2019-11-29; **Record Level:** type: Video; language: en; rightsHolder: Emiliano Cimoli
- **Type status:**Other material. **Occurrence:** individualID: MCMEC2018_Mertensiidae_sp_A_c; associatedMedia: http://morphobank.org/permalink/?P3993; **Taxon:** kingdom: Animalia; phylum: Ctenophora; class: Tentaculata; order: Cydippida; family: Mertensiidae; **Location:** continent: Antarctica; waterBody: McMurdo Sound; maximumDepthInMeters: 1; decimalLatitude: -77.637; decimalLongitude: 166.401; **Identification:** identifiedBy: Dhugal Lindsay; **Event:** samplingProtocol: NIKON D500 camera equipped with a TAMRON SP 90mm F2.8 Di Macro VC USD F017N lens; eventDate: 2018-11-27; **Record Level:** type: StillImage; language: en; rightsHolder: Emiliano Cimoli
- **Type status:**Other material. **Occurrence:** individualID: MCMEC2018_Mertensiidae_sp_A_d; associatedMedia: http://morphobank.org/permalink/?P3993; **Taxon:** kingdom: Animalia; phylum: Ctenophora; class: Tentaculata; order: Cydippida; family: Mertensiidae; **Location:** continent: Antarctica; waterBody: McMurdo Sound; maximumDepthInMeters: 1; decimalLatitude: -77.637; decimalLongitude: 166.401; **Identification:** identifiedBy: Dhugal Lindsay; **Event:** samplingProtocol: NIKON D500 camera equipped with a TAMRON SP 90mm F2.8 Di Macro VC USD F017N lens; eventDate: 2018-11-29; **Record Level:** type: StillImage; language: en; rightsHolder: Emiliano Cimoli
- **Type status:**Other material. **Occurrence:** individualID: MCMEC2018_Mertensiidae_sp_A_e; associatedMedia: http://morphobank.org/permalink/?P3993; **Taxon:** kingdom: Animalia; phylum: Ctenophora; class: Tentaculata; order: Cydippida; family: Mertensiidae; **Location:** continent: Antarctica; waterBody: McMurdo Sound; maximumDepthInMeters: 1; decimalLatitude: -77.637; decimalLongitude: 166.401; **Identification:** identifiedBy: Dhugal Lindsay; **Event:** samplingProtocol: Seabotix LBV-300 ROV equipped with a GoPro Hero 5; eventDate: 2018-12-01; **Record Level:** type: StillImage; language: en; rightsHolder: Emiliano Cimoli
- **Type status:**Other material. **Occurrence:** individualID: MCMEC2018_Mertensiidae_sp_A_f; associatedMedia: http://morphobank.org/permalink/?P3993; **Taxon:** kingdom: Animalia; phylum: Ctenophora; class: Tentaculata; order: Cydippida; family: Mertensiidae; **Location:** continent: Antarctica; waterBody: McMurdo Sound; maximumDepthInMeters: 1; decimalLatitude: -77.637; decimalLongitude: 166.401; **Identification:** identifiedBy: Dhugal Lindsay; **Event:** samplingProtocol: NIKON D500 camera equipped with a TAMRON SP 90mm F2.8 Di Macro VC USD F017N lens; eventDate: 2018-11-27; **Record Level:** type: StillImage; language: en; rightsHolder: Emiliano Cimoli

##### Distribution

first time observation.

##### Notes

Description of the family Mertensiidae: according to the key to Ctenophora by Licandro and Lindsay (2017), our observed specimens should be part of the Mertensiidae family, excluding the genus *Lampea*, based on the following morphological characters: Cydippida with presence of a pair of tentacles exiting aborally from opposite sides of the body, tentacles with tentilla, without oral lobes, tentacle sheath exits towards the aboral pole, tentacle attached at the aboral end of the tentacle bulb, type C internal canal structure. The body, however, is not laterally compressed. A recent phylogenetic study showed that the family Mertensiidae is non-monophyletic and, therefore, needs revision (Townsend et al. 2020). We provisionally place this species in the Mertensiidae *sensu lato*, which is also the current assignment of other such orphans, such as the Mertensiidae sp. of Wrobel and Mills (1998): cf. Figure 108. A record for a Mertensiidae sp. from the Danco coast of the Antarctic Peninsula, appears in Whelan et al. (2017) with the GenBank accession number MF599321. Figure 3 of the same paper apparently assigns this sequence to a 6.5 mm-long, whitish, elongated "Cydippida species Antarctica", but Table S1 in the supplementary material lists both a Mertensiidae sp. (64.65˚S, 62.396˚W) and a Cydippida sp. (63.439˚S, 55.453˚W) from Antarctica. Figure 2 in Whelan et al. (2017) refers to this sequence as Mertensiidae sp. Antarctica, while all other figures, including Figure 3 with the photograph, refer to this sequence as "Cydippida species Antarctica". Although the morphology of the animal in Figure 3 is vastly different from the undescribed species dealt with in the present paper, it is unclear whether the photograph assigned to the Mertensiid sequence in Whelan et al. (2017) is actually of that animal or whether it is a photo of something completely different. Photographs of "Mertensiid ctenophores" appear in Brueggeman (1998), but the tentacle bulbs lie parallel to the stomodeum in the first photo and the ridges are far more pronounced in the second photo compared to the present material. We, therefore, infer that other undescribed "mertensiid" species occur in the Ross Sea.

**Figure 14.**
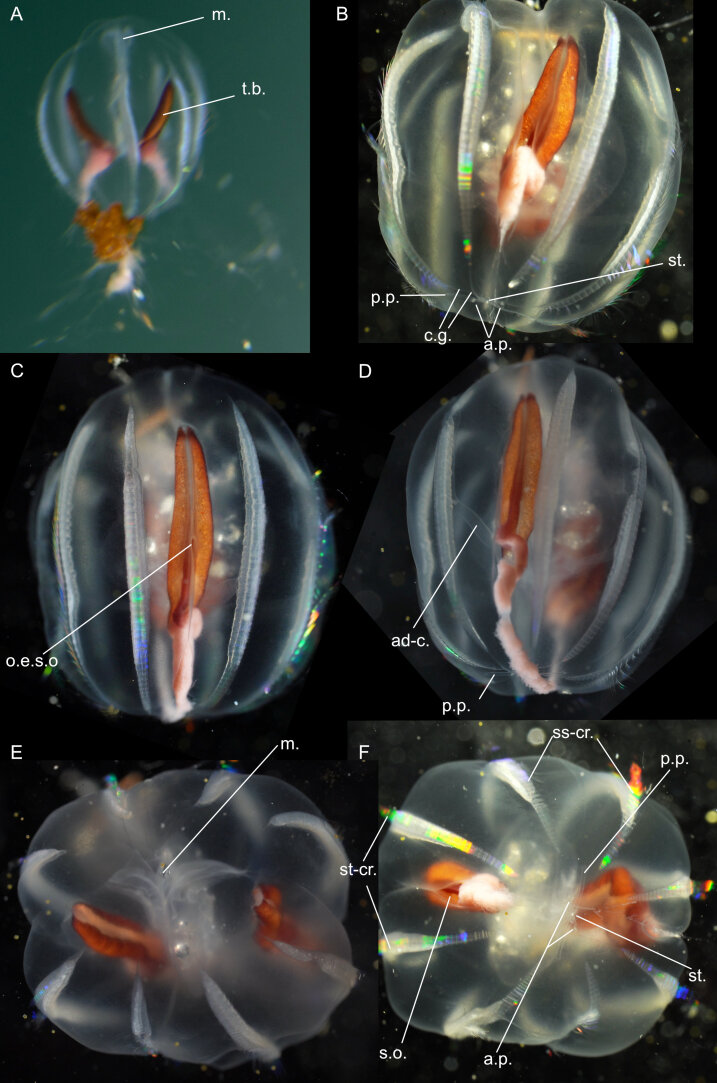
Mertensiidae sp. A. Lateral views (A-D), oral view (E) and aboral view (F) of specimens observed on 27/11/2018 (MCMEC2018_Mertensiidae_sp_A_c) (A) and 15/11/2019 (MCMEC2019_Mertensiidae_sp_A_a) (B-F). Abbreviations: ad-c., adradial canal; a.p., anal pore; c.g., ciliary grove; m., mouth; o.t.s.o., oral end opening of tentacle sheath; p.p., polar plate; s. o., tentacle sheath opening; st., statocyst; ss-cr., substomodeal comb row; st-cr., subtentacular comb row; t.b., tentacle bulb. C-H photos courtesy: E. Cimoli.

Comments on observed material: N = 4 in 2018 and N = 2 in 2019 (Fig. 14). Body nearly completely spherical, transparent; eight comb rows located equidistantly from each other, of same length, extending 90% of total body length, space between four comb plates ca. equal to width of comb plate, length of comb plates ca. three times inter-comb plate distance; type C internal canal structure (see Licandro and Lindsay 2017); tentacle bulb long (1/3 of total body length), orientated obliquely to the stomodeum (Fig. 14A) with aboral end located close to stomodeum, at one third from aboral end and oral end the furthest from the stomodeum, at one third of oral end, colour dark brown on the outside with salmon-coloured groove facing stomodeum; tentacles thick, dark red when leaving the aboral end of tentacle bulb, with light pink tentilla; tentacle sheath opening very long or situated within a deep lateral groove, running from close to polar plate (Fig. 14F) to about the height of the middle of the tentacle bulb; polar plate straight line; no ciliary ring encircling polar plate; two anal pores on both sides of statocyst; length of comb plates equal to six times the space between plates; stomodeum length up to aboral end of tentacle bulbs.

### Other Phyla

#### Mollusca

DD26E339-040E-5E26-B98D-EB878BAD5802

##### Notes

**Figure 15.**
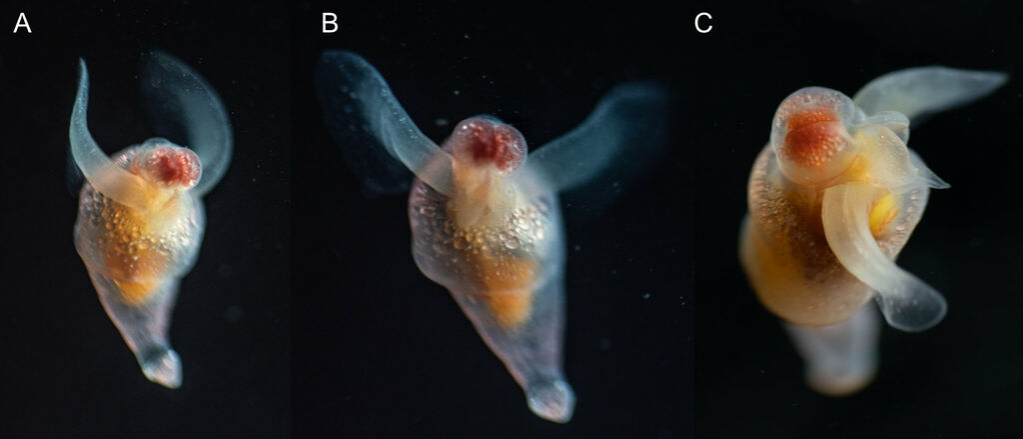
*Clionelimacinaantarctica*. **A**, **B.** Specimens observed on 29/11/2018; **C.** Specimen observed on 20/11/2019. Photos courtesy: E. Cimoli.

**Figure 16.**
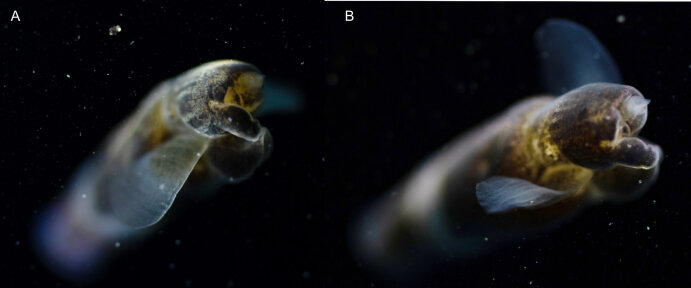
*Spongiobranchaeaaustralis*. **A**, **B.** Specimens observed on 29/11/2018. Photos courtesy: E. Cimoli.

**Figure 17.**
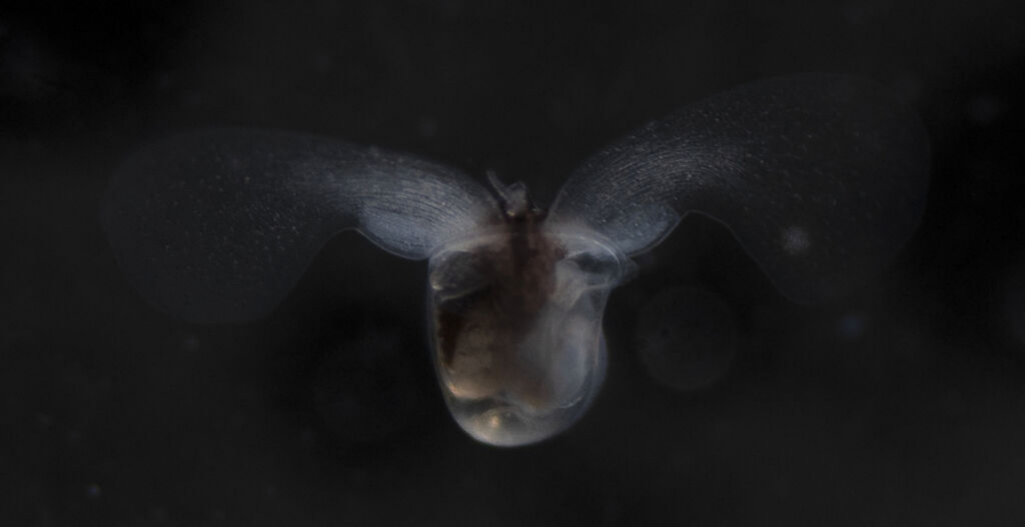
*Limacinahelicinaantarctica* specimen observed on 22/11/2019. Photo courtesy: E. Cimoli.

**Figure 18.**
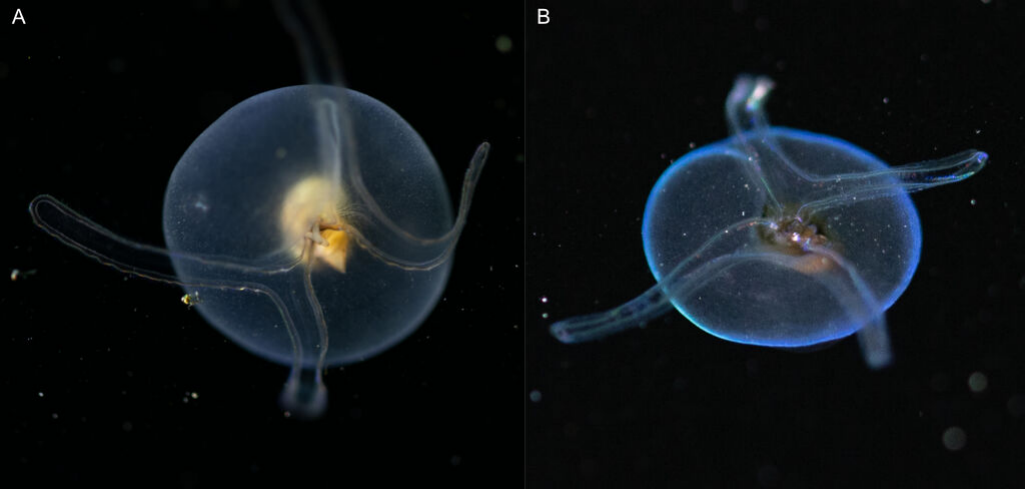
Gastropod larvae. **A.** Specimen observed on 16/11/2018; **B.** Specimen observed on 1/12/2018. Photos courtesy: E. Cimoli.

Mollusca was the third most-observed phylum (20% of all observations): *Clionelimacinaantarctica* (N = 2 in 2018, N = 1 in 2019) (Fig. 15), *Spongiobranchaeaaustralis* (N = 1 in 2018) (Fig. 16), *Limacinahelicinaantarctica* (N = 2 in 2019) (Fig. 17),and various Gastropod larvae (N = 3 in 2018, N = 2 in 2019) (Fig. 18).

#### Amphipoda

Latreille, 1816

B32230DB-33A4-535A-8919-FB7D15CACD8B

##### Notes

**Figure 19.**
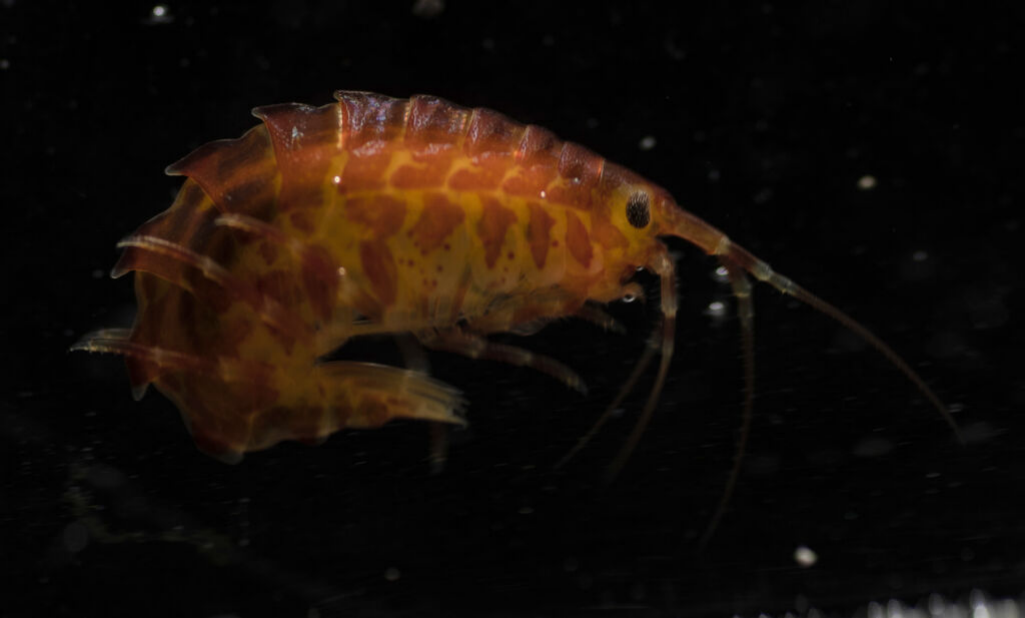
Eusirid amphipod specimen observed on 26/11/2019. Photo courtesy: E. Cimoli.

We observed a few amphilochid amphipods belonging to the family Eusiridae (N = 3 in 2018, N = 1 in 2019) (e.g. Fig. 19).

#### Syllidae

Grube, 1850

134A63E2-AD19-5BCE-B75A-300DE3BA4A9C

##### Notes

**Figure 20.**
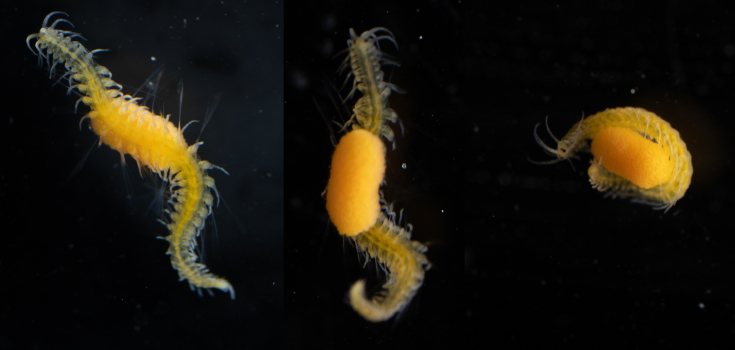
Syllidae polychaete carrying a large yellow egg sac observed on 22/11/2019. Photos courtesy: E. Cimoli.

We observed one syllid polychaete in 2019, carrying a large yellow egg sac (Fig. 20).

## Analysis

### Synopsis of observed species

A total of 49 individuals were observed during the summer of 2018 (N = 25) and 2019 (N = 24). The majority of observed specimens belonged either to the phylum Cnidaria (36.7%) or Ctenophora (30.6%), whereas the remaining observed phyla, namely Mollusca (22.4%), Arthropoda (8.1%) and Annelida (2.0%), were less represented. The observed species are summarised in Table 2.

## Discussion

In this study, we conducted an *in situ* /*in aqua* optical survey of gelatinous zooplankton from under the ice in the McMurdo Sound, Antarctica. Our study represents the first formal optics-based survey of gelatinous zooplankton in the Ross Sea and the first study to use *in situ* /*in aqua* observations to describe taxonomic and a few trophic and behavioural characteristics of gelatinous zooplankton from the Southern Ocean. The Ross Sea has seen numerous net sampling surveys of gelatinous zooplankton in the past (e.g. Foster 1989, Larson and Harbison 1990, Browne 1910). Nevertheless and despite the small geographic (one sampling location) and temporal (two times, three weeks each) scales of our study, we reported: new undescribed morphological traits for all observed gelatinous zooplankton species (seven medusae and four ctenophore species) and first time observations in the Ross Sea for one Leptothecata and four ctenophore species (Table 2). Furthermore, along with the photography and videography, we prepared a Common Objects in Context (COCO) dataset, so that this study is the first to provide a taxonomist-ratified image training set for future machine-learning algorithm development concerning Southern Ocean gelatinous zooplankton species.

Our study demonstrates how valuable optical *in situ* observations are to investigate gelatinous zooplankton. Nonetheless, our study also encountered a few limitations. For example, despite most of the studied species being transparent, the observation of internal morphological characters is difficult without the collection and dissection of the specimens. A second limitation lies in the identification of certain species, based on morphological traits alone, especially for genera with numerous species, such as *Beroe* spp., where the type specimens are no longer extant. For one leptomedusan and three ctenophore species, their morphology did not match that of any species previously reported from the Southern Ocean. They could potentially be undescribed species, although this needs to be confirmed through DNA barcoding of all the described species from their type localities and further morphological comparisons. The future of gelatinous zooplankton studies lies in the integration of different methodologies, including appropriate collection methods, optical survey tools and molecular genetic comparisons.

## Supplementary Material

4A2E024C-6532-5178-82D6-BB0CB26B8766 10.3897/BDJ.9.e69374.suppl1 Supplementary material 1 Permission letter to reuse Fig. 2D (Schuchert 1996) Data type PDF File: oo_573830.pdf https://binary.pensoft.net/file/573830 Dr. Michelle Kelly

5E8DCF32-F4EA-5BE8-BE25-EC437433D7BD 10.3897/BDJ.9.e69374.suppl2 Supplementary material 2 Springer Nature Licence to reuse Fig. 3A (Larson and Harbison 1990) Data type PDF Brief description Licence number: 5119250419357 File: oo_573831.pdf https://binary.pensoft.net/file/573831 Dr. Gerlien Verhaegen

XML Treatment for Koellikerina maasi

XML Treatment for Leuckartiara brownei

XML Treatment for Solmundella bitentaculata

XML Treatment for Leptomedusa sp. A

XML Treatment for Leptomedusa sp. B

XML Treatment for Pyrostephos vanhoeffeni

XML Treatment for Diplulmaris antarctica

XML Treatment for Beroe

XML Treatment for Beroe sp. A

XML Treatment for Beroe sp. B

XML Treatment for Callianira cristata

XML Treatment for "fam. Mertensiidae" sp. A

XML Treatment for Mollusca

XML Treatment for Amphipoda

XML Treatment for Syllidae

## Figures and Tables

**Figure 10a.**
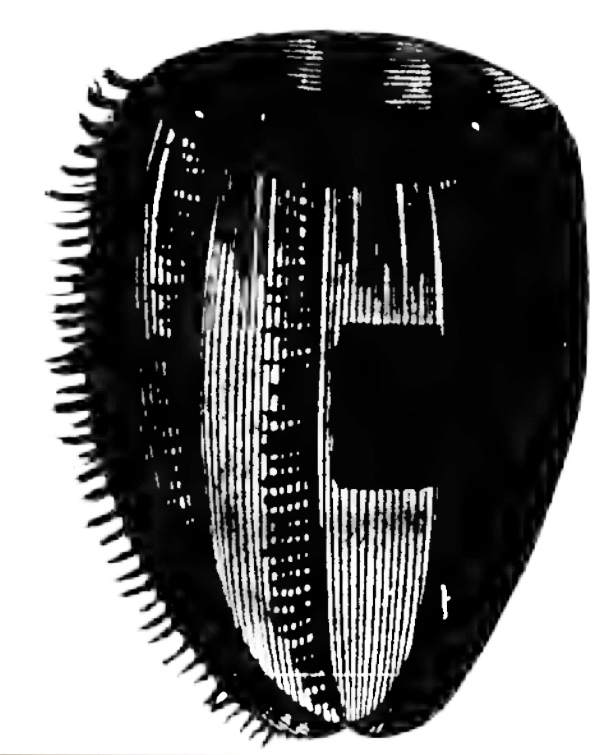
*Beroeovatus* Bosc, 1802, drawing (modified from Bosc 1802).

**Figure 10b.**
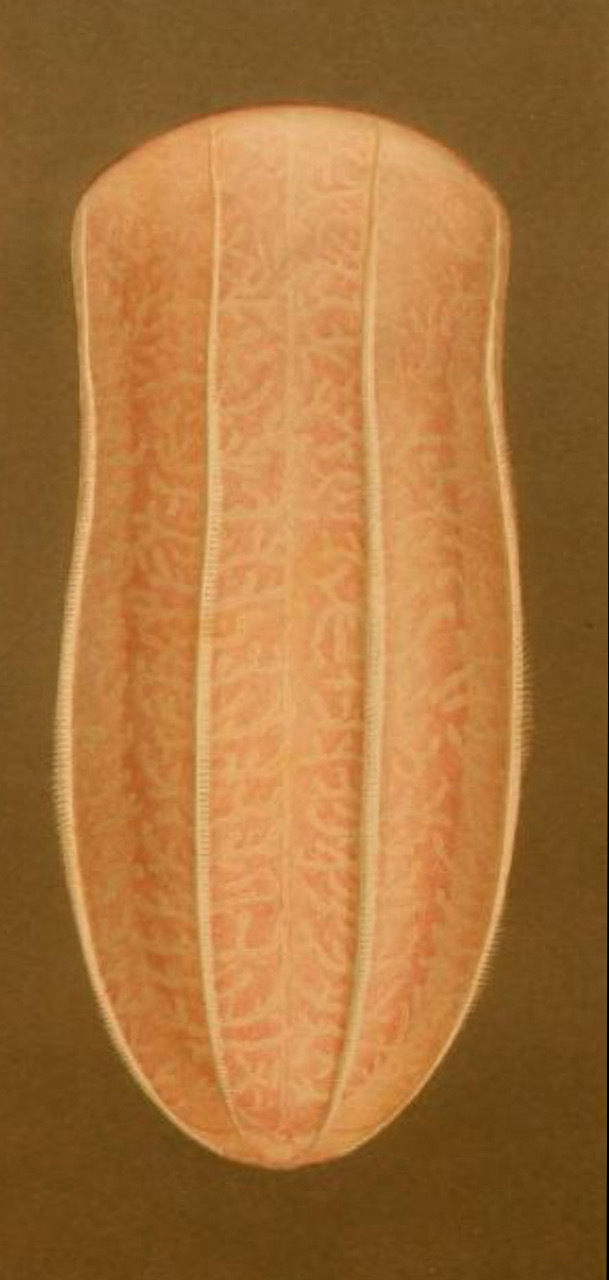
*Beroeovata* sensu Chun, 1880, drawing of an adult specimen from the Gulf of Naples, Mediterranean Sea (modified from Chun 1880).

**Figure 10c.**
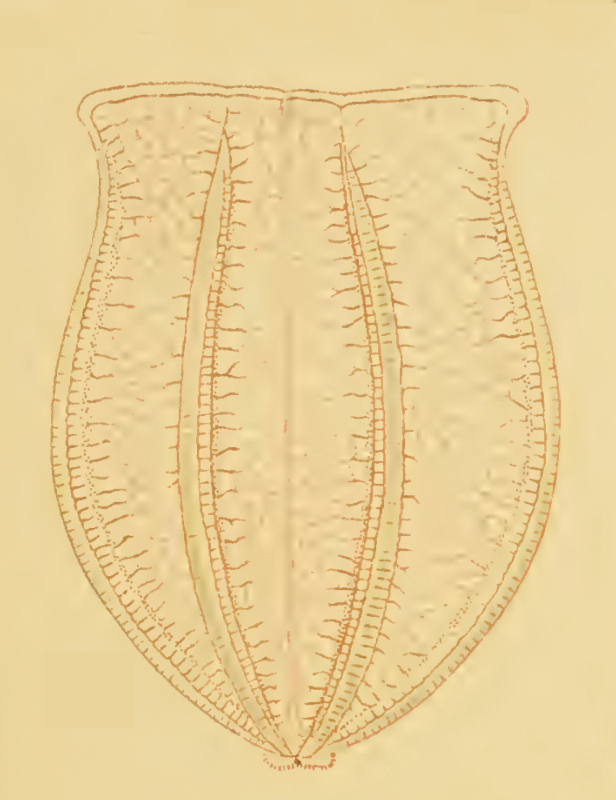
*Beroeovata* sensu Mayer, 1912 drawing of a specimen from St. Mary’s River, Maryland (modified from Mayer 1912).

**Figure 10d.**
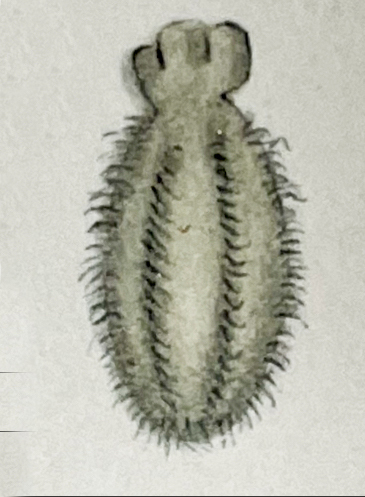
*Beroecompacta* Moser, 1909 drawing (length 2.5 mm) from Gauss Station, Antarctica (modified from Moser 1909).

**Figure 11a.**
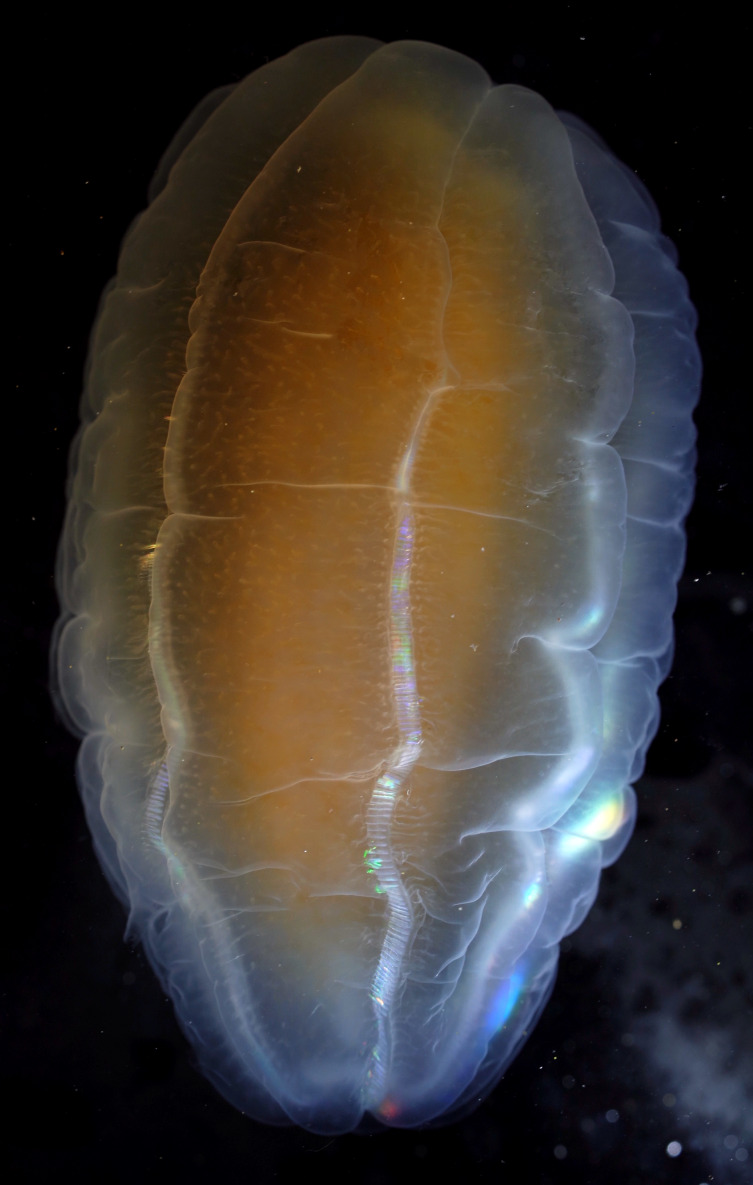
Adult specimen observed on 16/11/2019 (MCMEC2019_Beroe_sp_A_a)

**Figure 11b.**
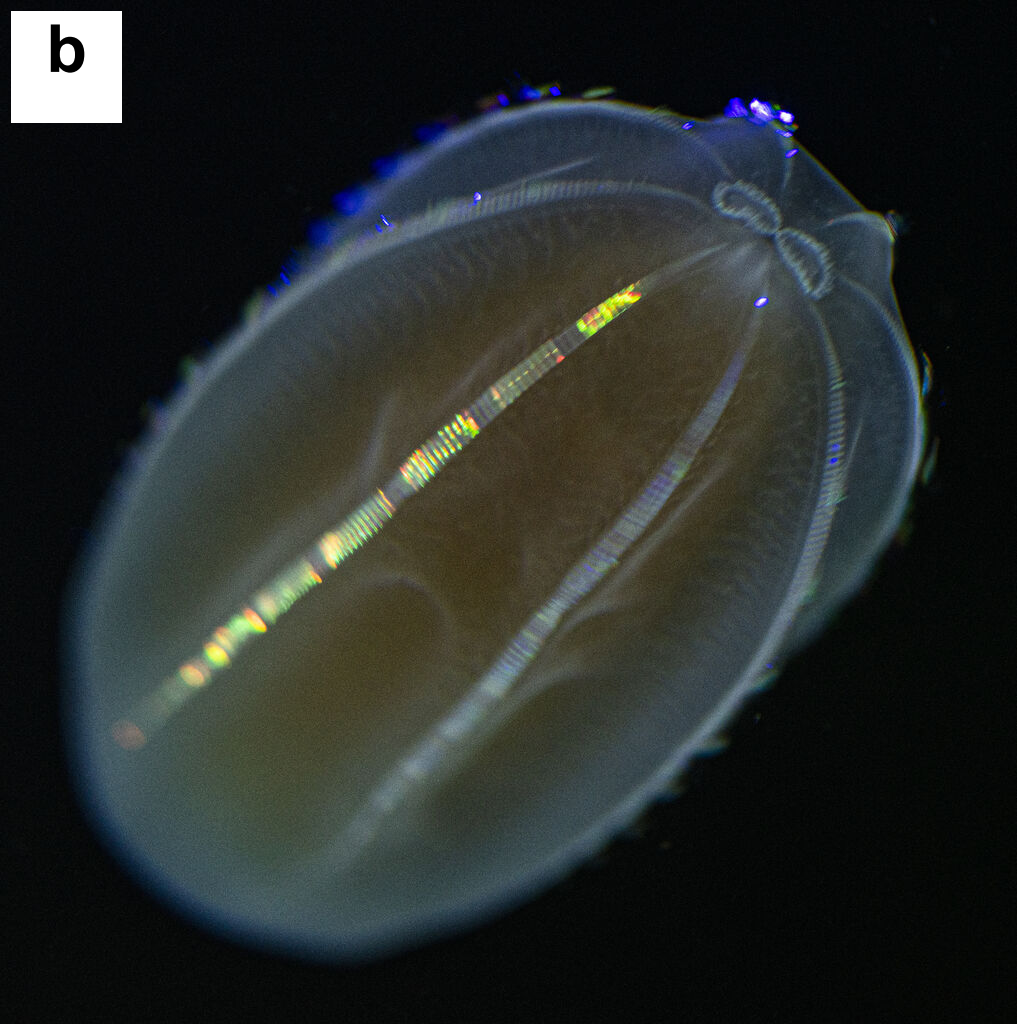
Adult specimen in apico-lateral view observed on 16/11/2018 (MCMEC2018_Beroe_sp_A_c)

**Figure 11c.**
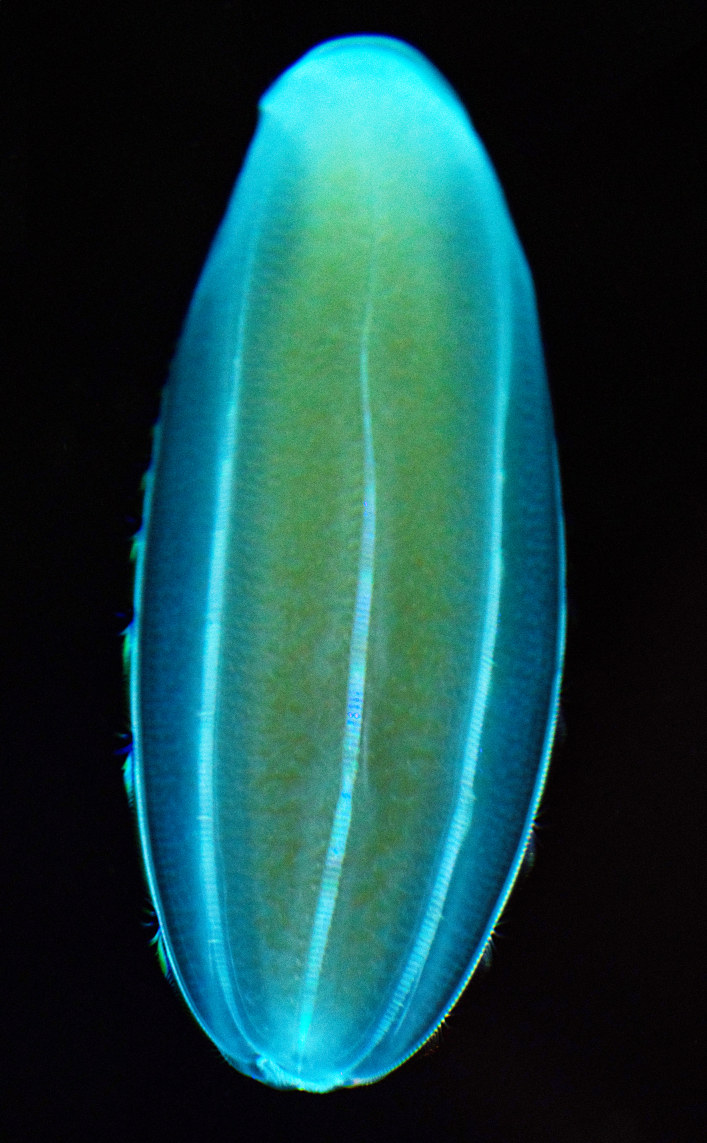
Adult specimen in lateral view observed on 16/11/2018 (MCMEC2018_Beroe_sp_A_c)

**Figure 11d.**
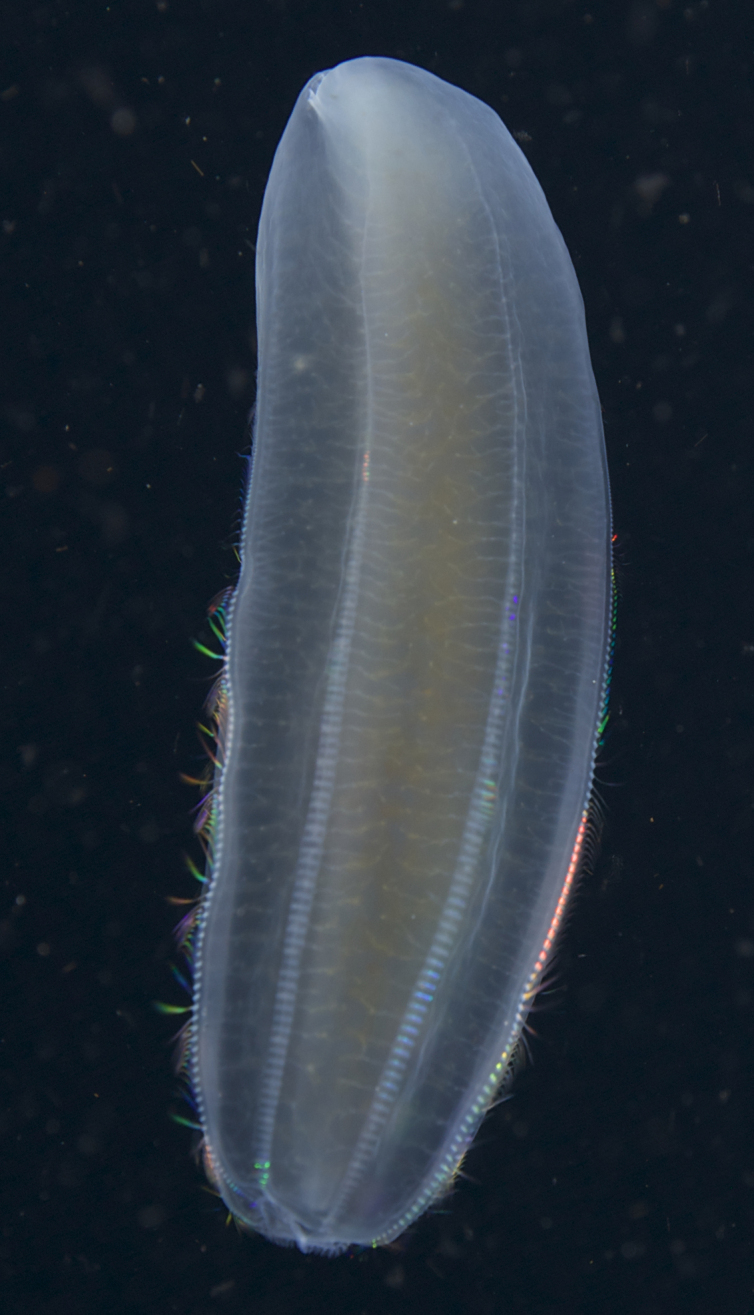
Adult specimen observed at Little Razorback Island, Ross Sea, on 02/12/2010 (LRISH2010_Beroe_sp_A_g).

**Figure 11e.**
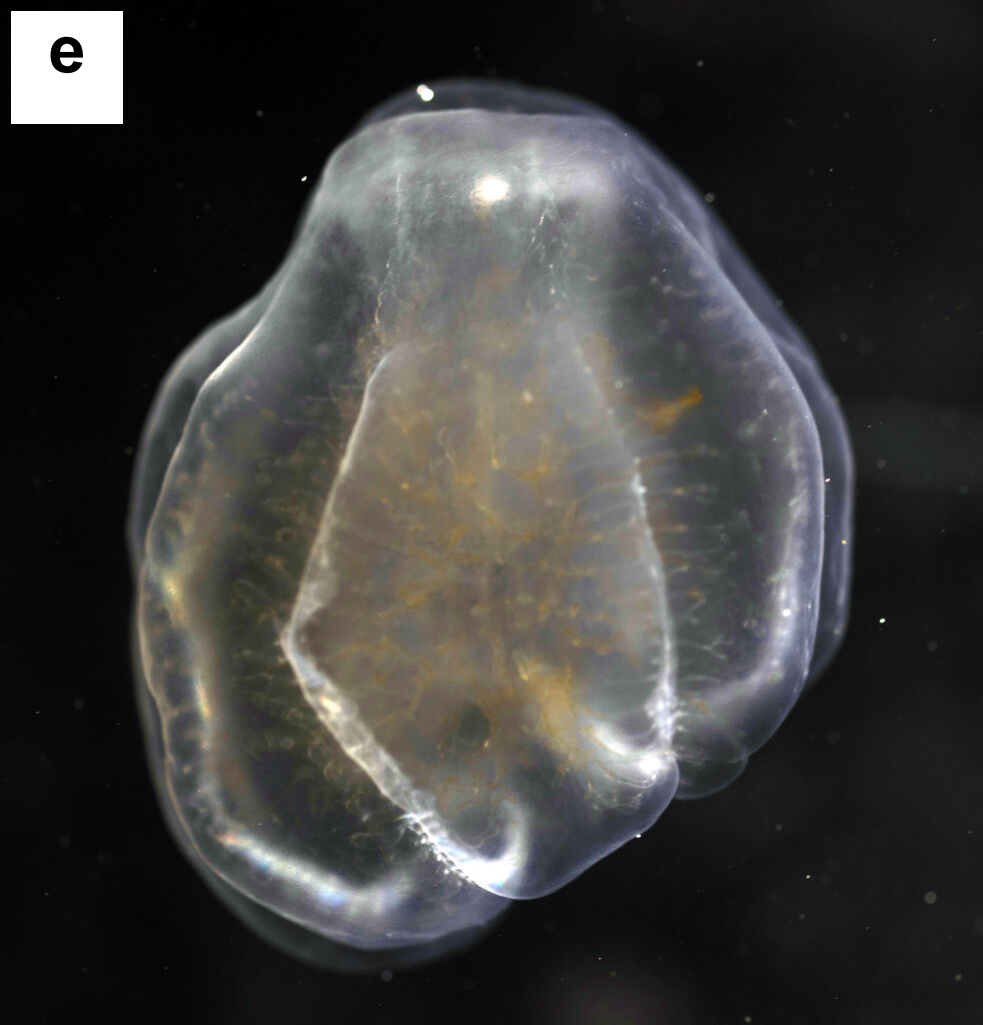
Juvenile specimen MCMEC2019_Beroe_sp_A_b in stomodeal view observed on 30/11/2019.

**Figure 11f.**
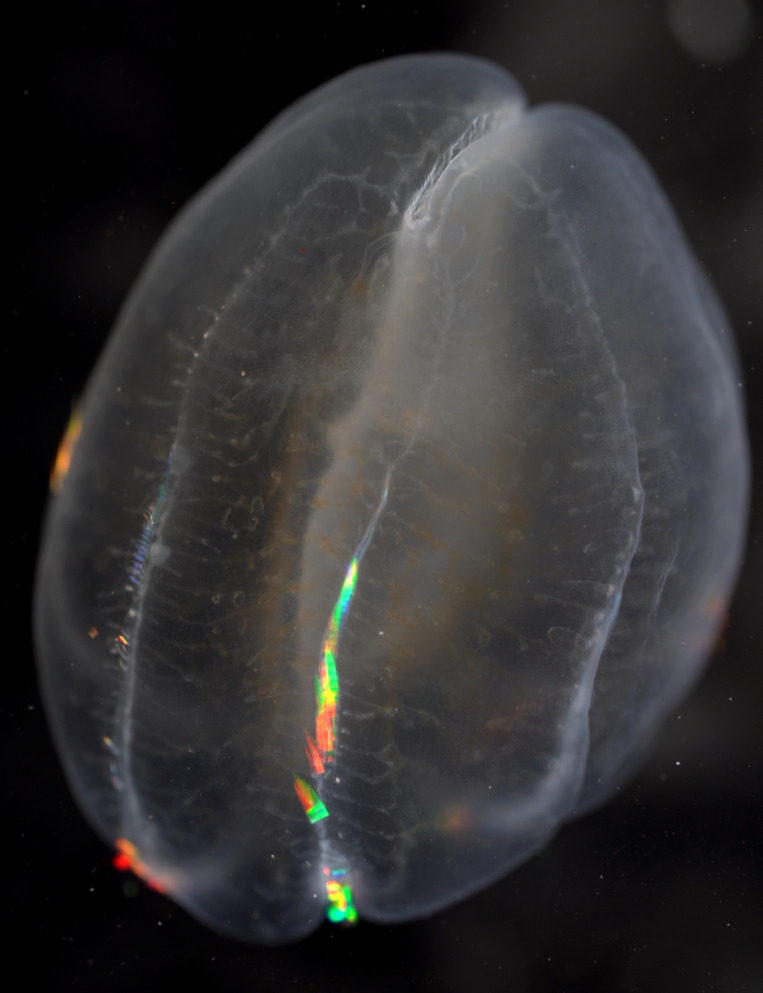
Juvenile specimen MCMEC2019_Beroe_sp_A_b observed on 30/11/2019.

**Table 1.** Morphological characteristics of *Beroe* species that have been reported for the Southern Ocean (the compared characters were chosen, based on those that were evident in our filmed specimens).

**Species**	**Body length (L) adults (mm)**	**Body shape**	**Comb row length vs. body length adult**	**Inter-comb plate distance**	**Branching from meridional canals**	**Colour**	**Illustration**	**Type locality**
*B.compacta* Moser, 1909	2.5	cylindrical	whole body length (based on drawing)	short	/	opaque, shimmering yellowish between white comb rows	Fig. 10d	Gauss Station, Antarctica
*B.cucumis* Fabricius, 1780	/	oblong shape, elongated towards the extremities	whole body length	/	/	whitish with pink/red dots	/	Greenland
*B.ovatus* Bosc, 1802	/	oval	whole body length (based on drawing)	same as comb plate width (based on drawing)	/	transparent with nine uncoloured comb rows	Fig. 10a	“all seas”
*B.ovatasensu* Chun, 1880	< 160	body elongated, cylindrical, not very noticeably compressed, gradually tapering towards the aboral pole in a semicircular arc	3/4 (based on drawing)	short (space between three comb plates ca. equal to width of comb plate, based on drawing)	numerous diverticula, no anastomoses	young transparent; adults during period of increased reproduction pink or bright red, otherwise unpigmented, grey-white or light transparent red	Fig. 10c	Gulf of Naples, Mediterranean Sea
*B.ovatasensu* Mayer, 1912	70-115	mitre-shaped with lateral compression very marked and mouth a wide-gaping slit	3/4 (1/2 in juveniles)	wide (space between two comb plates ca. equal to width of comb plate, based on drawing)	loose network of numerous diverticula with few anastomoses	dull-milky (in Florida) to highly coloured, with deep pink or reddish-brown canals (in northern waters as in Chesapeake Bay)	Fig. 10b	Atlantic Coast of North America
*Beroe* sp. A	/	oval (body length ca. 2.4 times body width)	ca. 2/3 of body length	short (space between four comb plates ca. equal to width of comb plate)	diverticula without anastomoses	brownish-orange stomodeum and diverticula	Fig. 11	Mc Murdo Sound, this study
*Beroe* sp. B	ca. 35	oval (body length ca. 1.5 times body width)	ca. 1/4 of body length	short (space between 5 comb plates ca. equal to width of comb plate)	no diverticula	transparent to milky white	Fig. 12	Mc Murdo Sound, this study

**Table 2.** Summary of observed specimens

**Phylum**	**Taxa**		**Species**	**N (2018)**	**N (2019)**	**First time report for the Ross sea**?	**Figures**
Cnidaria	Hydrozoa (class)	Anthoathecata (order)	* Koellikerina maasi *	2	1	no	Fig. 2
			* Leuckartiara brownei *	2	1	no	Fig. 3
		Leptothecata (order)	Leptomedusa sp. A	0	1	yes	Fig. 5
			Leptomedusa sp. B	0	1	no (if our proposed species assignment is correct)	Fig. 6
		Narcomedusae (order)	* Solmundella bitentaculata *	1	0	no	Fig. 4
		Siphonophorae (order)	* Pyrostephos vanhoeffeni *	4	2	no	Fig. 7
	Scyphozoa (class)	Semaeostomeae (order)	* Diplulmaris antarctica *	0	3	no	Figs. 8-9
Ctenophora	Beroida (order)		*Beroe* sp. A	3	4	yes (previously only images erroneously assigned to *Beroecucumis* were published, online)	Fig. 10
			*Beroe* sp. B	0	1	yes	Fig. 11
	Cydippida (order)		* Callianira cristata *	0	1	yes (previously only images assigned to Mertensiidae were published, online)	Fig. 12
			Mertensiidae sp. A	4	2	yes	Fig. 13
Mollusca	Pteropoda (order)		* Clione limacina antarctica *	2	1	no	Fig. 14
			* Spongiobranchaea australis *	1	0	no	Fig. 15
			* Limacina helicina antarctica *	0	2	NA	Fig. 16
	incertae sedis		Gastropoda larvae	3	2	NA	Fig. 17
Arthropoda	Amphipoda (order)		Eusiridae	3	1	NA	Fig. 18
Annelida	Polychaeta (class)		Syllidae	0	1	NA	Fig. 19
			Total	25	24		
