# Supplementary material for: Life beneath the ice: jellyfish and ctenophores from the Ross Sea, Antarctica, with an image-based training set for machine learning
Source: Biodivers Data J. 2021 Aug 16;9:e69374. doi: 10.3897/BDJ.9.e69374 (PMC8382665; doi:10.3897/BDJ.9.e69374)
Supplement: Supplementary material 1 — Permission letter to reuse Fig. 2D (Schuchert 1996) [file bdj-09-e69374-s001.pdf]

NIWA
Taihoro Nukurangi

3 August 2021

Dear Gerlien Verhaegen

Thank you for your request to reproduce a figure from one of our monographs.

The *NIWA Biodiversity Memoir (NBM) Series* is licensed under the Creative Commons Attribution-NonCommercial-NoDerivs 3.0 Unported License. To view a copy of this license, visit http://creativecommons.org/licenses/by-nc-nd/3.0/. As such, you are free to copy and redistribute the material in any medium or format under the following terms:

(1) You must give appropriate credit to the NBM series and indicate if changes were made to the figure, in the caption
(2) You may not use the material for commercial purposes
(3) If you remix, transform, or build upon the material, you may not distribute the modified material

This letter grants you permission to modify and/or reproduce images and/or other materials previously published in the *NIWA Biodiversity Memoir (NBM) Series* (previously known as the New Zealand Oceanographic Institute Memoir, and the New Zealand Department of Scientific and Industrial Research Bulletin).

(1) Please list the *NIWA Biodiversity Memoir* from which the materials are requested: Schuchert, P. (1996) The marine fauna of New Zealand: Athecate hydroids and their medusae (Cnidaria, Hydrozoa). New Zealand Oceanographic Institute Memoir 106, 147 pp.
(2) Please list the requested materials below: NBM 106, Fig. 22
(3) Please provide a credit line in your caption or acknowledgements, indicating if any changes have been made to the figure, as per the following example: Figure 22 is reproduced from Schuchert, P. (1996) *NIWA Biodiversity Memoir* 106, 1–147, with permission from Dr Michelle Kelly, Editor, NIWA Biodiversity Memoir Series.

With kindest regards

Michelle Kelly

Michelle Kelly, PhD, DSc (Science)
Managing editor - NIWA *Marine Biodiversity Memoir* Series

G. Verhaegen

Dr. Gerlien Verhaegen
Japan Agency for Marine-Earth Science and Technology (JAMSTEC)

National Institute
of Water & Atmospheric
Research Ltd (NIWA)

41 Market Place
Viaduct Harbour
Auckland 1010
Private Bag 99940
Newmarket
Auckland 1149

P: +64 9 375 2050
enquiries@niwa.co.nz
www.niwa.co.nz
